# A unified framework for molecular property prediction based on hierarchical multi-granularity molecular representation learning

**DOI:** 10.1093/bioinformatics/btag600

**Published:** 2026-08-10

**Authors:** Xing Zhao, Xianlai Chen, Yunbo Wang, Ying An, Haochen Zhao

**Affiliations:** Big Data Institute, Central South University, Changsha 410083, China; Big Data Institute, Central South University, Changsha 410083, China; Big Data Institute, Central South University, Changsha 410083, China; Big Data Institute, Central South University, Changsha 410083, China; Big Data Institute, Central South University, Changsha 410083, China

## Abstract

**Motivation:**

Molecular property prediction is a fundamental task in drug discovery and plays a key role in accelerating the identification of promising candidates. Existing methods have primarily focused on atom–bond information for molecular representation learning. However, atoms and bonds are often not explicitly organized as coupled learnable entities, and prior knowledge such as functional motifs and global molecular attributes remains largely underutilized. In addition, they lack effective mechanisms to enable interaction and fusion across different structural granularities. Consequently, the resulting representations often capture only partial chemical semantics.

**Results:**

To address these issues, we propose HMG-MRL, a unified framework for hierarchical multi-granularity molecular representation learning. This framework systematically integrates domain knowledge across three granularities: fine-grained atom–bond information, medium-grained functional motifs, and coarse-grained molecular attributes. Specifically, we introduce an atom–bond bipartite graph modeling approach that treats atoms and bonds as explicit learnable node types and jointly models atom–atom, atom–bond, and bond–bond local interactions within a unified propagation framework. In parallel, we integrate multiple substructure decomposition strategies to construct a diverse motif vocabulary and design a Motif Transformer that employs self-attention to capture global interactions among motifs. Moreover, we design a cross-granularity communication module to facilitate information exchange across granularities. Extensive experiments on nine public benchmark datasets show that HMG-MRL achieves competitive predictive performance. Case studies further indicate that the model can reveal key molecular components across different granularities, with its diverse substructure decomposition strategies offering complementary structural patterns.

**Availability and implementation:**

The data and code of HMG-MRL are available at https://github.com/AKZstar/HMG-MRL.

## 1 Introduction

Accurate prediction of bioactivity and ADMET properties (absorption, distribution, metabolism, excretion, and toxicity) in the early stages of drug development is critical for efficiently eliminating compounds with poor drug-likeness, thereby accelerating development cycles and mitigating financial risks (Zhao *et al.* 2023, 2025a, Guo *et al.* 2025, Zhang *et al.* 2025a, Zheng *et al.* 2025). Early studies predominantly used traditional machine learning coupled with handcrafted features exemplified by molecular descriptors (Deng *et al.* 2023, Kuang *et al.* 2024). However, these approaches suffer from limited representational capacity and heavy reliance on domain expertise, hindering their ability to capture complex chemical semantics and limiting model performance (Li *et al.* 2022).

As molecules are naturally structured as graphs, Graph Neural Networks (GNNs) (Tang *et al.* 2023, Khemani *et al.* 2024, Zhang *et al.* 2025b) have emerged as a leading paradigm for molecular property prediction, owing to their ability to integrate graph topology with node attributes for expressive feature learning. For example, AttentiveFP (Xiong *et al.* 2020) and ResGAT (Nguyen-Vo *et al.* 2024) perform atom-centered message passing and leverage attention mechanisms to enhance atomic embeddings as well as molecular representations for property prediction. DMPNN (Yang *et al.* 2019), TrimNet (Li *et al.* 2021), and line-graph formulations such as the Line Graph Transformer in KPGT (Li *et al.* 2023) organize message passing mainly through directed bonds, bond-aware messages, or bond-derived nodes. CMPNN (Song *et al.* 2020) updates node and edge representations through a directed and sequential communication pipeline, while ABT-MPNN (Liu *et al.* 2023) introduces bond-level and atom-level attention in a staged bond-to-atom architecture. Although these methods model atom- and bond-related dependencies in different ways, atoms and bonds are generally not maintained as two explicit learnable node types whose updates jointly incorporate homogeneous and heterogeneous neighborhood information within each layer. This limits the direct characterization of coupled local chemical environments, where atom types, bond attributes, and local topological arrangement need to be integrated through atom–atom, atom–bond, and bond–bond interactions.

Existing studies have shown that modeling only at the atomic and bond levels (Deng *et al.* 2023, Kuang *et al.* 2024) is insufficient to fully capture molecular properties. Higher-order chemical motifs [e.g. functional groups and BRICS fragments (Degen *et al.* 2008)] and their interactions often exert a critical influence on molecular behavior. This has motivated a line of work that incorporates motif information into molecular representations (Han *et al.* 2023, Jiang *et al.* 2023, Zhang *et al.* 2021). For example, GraphFP (Luong and Singh 2023) employs dual GNN encoders for molecular and fragment graphs, respectively, and utilizes contrastive pretraining to effectively capture both local patterns and higher-order connectivity within molecules. By integrating fragments derived from the BRICS algorithm with atomic-level features, researchers have developed hierarchical molecular representation models (Zhu *et al.* 2023, Han *et al.* 2025) for property prediction. To further exploit scaffold information for molecular property prediction, a multi-channel pretraining framework (Wan *et al.* 2025) incorporating a scaffold contrastive distance learning task has also been proposed. However, these methods depend on a single and fixed substructure perspective, which severely restricts motif diversity and may overlook property-relevant motifs, and they also lack explicit modeling of global interactions among motifs. In addition, prior studies have shown that molecular properties ultimately arise from the intricate interplay across multiple structural granularities (Deng *et al.* 2023, Kuang *et al.* 2024, Xiang *et al.* 2025, Zhao *et al.* 2026). However, most current approaches overlook explicit cross-granularity information exchange and rely solely on late fusion at the molecular level (Ma *et al.* 2022, Liu *et al.* 2023, Zhu *et al.* 2023).

To address these limitations, we propose HMG-MRL, a unified framework for hierarchical multi-granularity molecular representation learning. This framework systematically integrates domain knowledge across three granularities: fine-grained atom–bond information, medium-grained functional motifs, and coarse-grained molecular attributes. Specifically, we introduce an atom–bond bipartite graph modeling approach that maintains atoms and bonds as two explicit learnable node types and synchronously updates their representations by aggregating homogeneous and heterogeneous neighborhood information. This design enables atom–atom, atom–bond, and bond–bond local interactions to be modeled within a unified propagation framework, providing an explicit inductive bias for characterizing coupled local chemical environments involving atomic identities, bond attributes, and local molecular topology. In parallel, we integrate multiple substructure decomposition strategies to construct a diverse motif vocabulary and design a Motif Transformer to capture global interactions among functional motifs. Furthermore, we propose a cross-granularity communication module to facilitate information exchange across granularities, together with a cross-granularity alignment loss to enhance semantic consistency between the atom–bond and motif views. Finally, representations from all granularities are adaptively fused via an attention mechanism, yielding a unified and expressive molecular representation. Experimental evaluation on nine public benchmark datasets further demonstrates the competitive performance of HMG-MRL across both classification and regression tasks. Ablation results further confirm that its performance gains rely on both multi-granularity information and cross-granularity communication, as neither component alone is sufficient. Moreover, case studies show that HMG-MRL can reveal key molecular components across different granularities, and that its diverse substructure decomposition strategies provide complementary structural patterns.

## 2 Materials and methods

### 2.1 Datasets

We evaluate models on nine widely used benchmark datasets from MoleculeNet (Wu *et al.* 2018), encompassing both molecular property classification and regression tasks. The classification benchmarks include BACE, BBBP, SIDER, ClinTox, HIV, and Tox21, while ESOL, FreeSolv, and Lipophilicity serve as regression datasets. These datasets collectively span a broad spectrum of bioactivity, physiological, toxicity, and physicochemical endpoints, providing a comprehensive evaluation of the proposed framework. Detailed descriptions of each dataset can be found in Section 1, available as supplementary data at *Bioinformatics* online. For each dataset, we divide the data into training, validation, and test sets (8:1:1) using scaffold-based splitting (Yang *et al.* 2019). Five independent runs with different random seeds are conducted, and the mean and standard deviation are reported.

### 2.2 Overview of HMG-MRL

**Figure 1:**
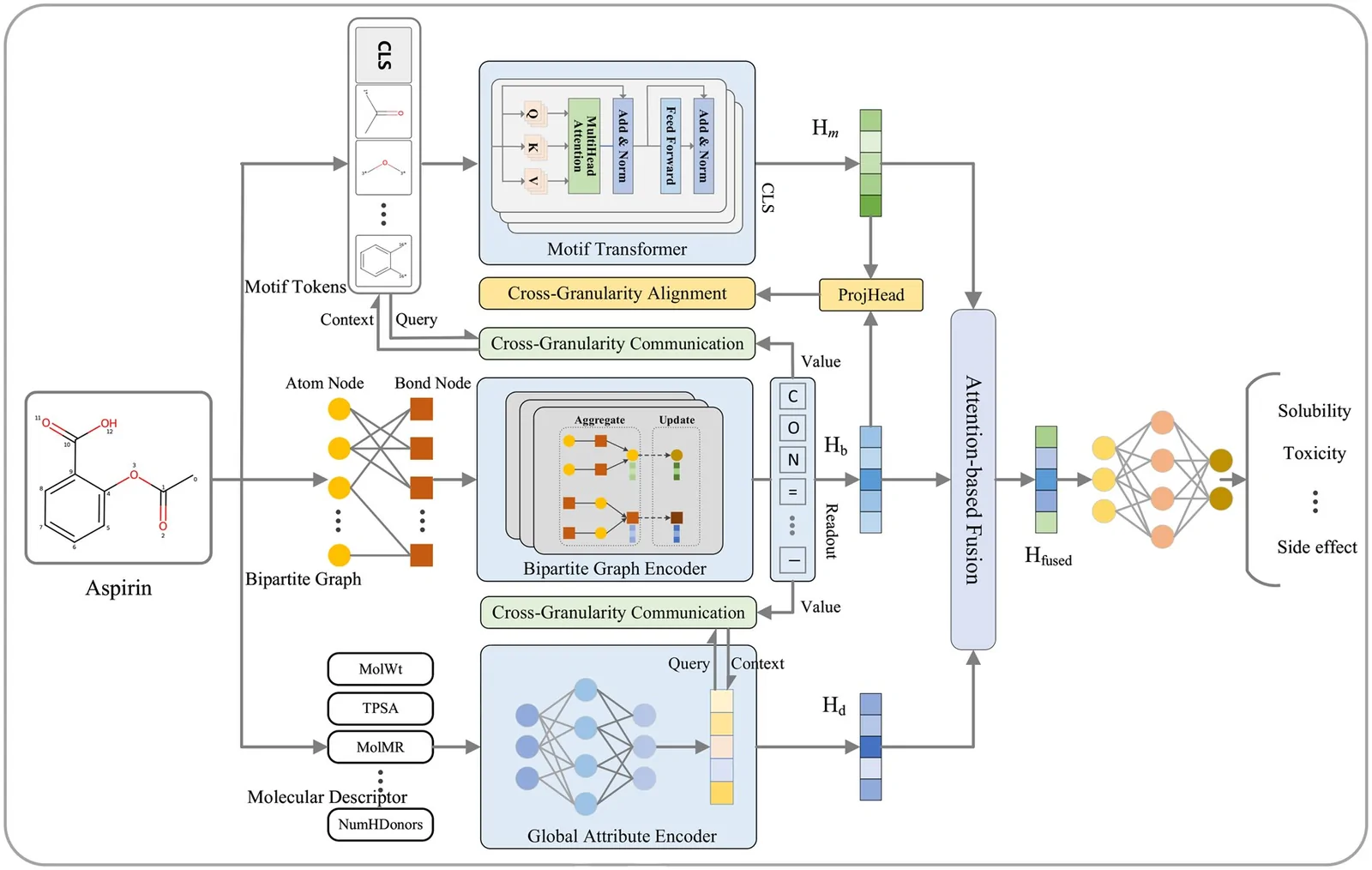
Overview of the HMG-MRL framework. HMG-MRL comprises three encoders—the Bipartite Graph Encoder, the Motif Transformer, and the Global Attribute Encoder—which capture domain knowledge at different granularities to generate hierarchical molecular representations ($H_{b}$, $H_{m}$, $H_{d}$). A cross-granularity communication module enables contextual information extraction from fine-grained atomic features under the guidance of global attributes and functional motifs, while a contrastive loss is applied to align motif and bipartite graph representations. Finally, all three representations are adaptively fused via an attention-based module to form the final molecular representation ($H_{\mathrm{fused}}$) for property prediction.

HMG-MRL comprises three encoders (Fig. 1)—the Bipartite Graph Encoder, the Motif Transformer, and the Global Attribute Encoder—each capturing domain knowledge at a distinct structural granularity and producing a corresponding molecular representation. During the encoding process, a cross-granularity communication module is designed to extract contextual information from atomic features under the guidance of motif information and molecular attributes. Moreover, to ensure semantic consistency between the atom–bond and motif views, we introduce a cross-granularity alignment loss. The resulting granularity-specific representations are then integrated through an attention-based fusion module to generate a unified molecular representation for property prediction.

#### 2.2.1 Bipartite Graph Encoder

To explicitly and jointly model atom–atom, atom–bond, and bond–bond local interactions, we propose an atom–bond bipartite graph modeling approach that maintains atoms and bonds as two explicit learnable node types. Formally, given a molecule, we define a bipartite graph $G=(V_{a},V_{b},E)$, where $V_{a}$ and $V_{b}$ denote the sets of atom nodes and bond nodes, respectively; E denotes the set of edges connecting atoms and bonds. For an atom node $i\in V_{a}$, its neighbors include (i) directly connected bonds, $N_{b}(i)$, and (ii) atoms connected via bonds, $N_{a}(i)$. Similarly, for a bond node $p\in V_{b}$, its neighbors include (i) the two atoms it connects, $N_{a}(p)$, and (ii) other bonds incident to these atoms, $N_{b}(p)$.

Building on this bipartite formulation, we develop an encoder that jointly updates atom and bond embeddings through iterative message passing. Specifically, the initial features of the two types of nodes are mapped into their corresponding embedding spaces as follows:


(1) $$h_{i}^{\left(a,0\right)}={\text{Embed}}_{a}(x_{i}^{a}),\;h_{p}^{\left(b,0\right)}={\text{Embed}}_{b}(x_{p}^{b})$$

Here, $x_{i}^{a}$ denotes the initial feature vector of atom *i*, and $x_{p}^{b}$ denotes the initial feature vector of bond *p*, both extracted using RDKit (see Section 2, available as supplementary data at *Bioinformatics* online). ${\text{Embed}}_{a}(\cdot )$ and ${\text{Embed}}_{b}(\cdot )$ denote two independent multi-layer perceptron (MLP)-based embedding layers. The resulting vectors $h_{i}^{\left(a,0\right)}$ and $h_{p}^{\left(b,0\right)}$ serve as the initial atom and bond embeddings, respectively. Then, the embeddings of nodes are updated by aggregating information from both homogeneous and heterogeneous neighbors. In the *l*-th layer, the contextual representations of atom *i* and bond *p* are computed separately, as follows:


(2) $$c_{i}^{\left(a,l\right)}=\sum_{j\in N_{a}(i)}\left(W_{a}h_{j}^{\left(a,l\right)}+W_{b}h_{\eta (i,j)}^{\left(b,l\right)}\right)$$

(3) $$c_{p}^{\left(b,l\right)}=\sum_{q\in N_{b}(p)}\left(W_{b}h_{q}^{\left(b,l\right)}+W_{a}h_{\xi (p,q)}^{\left(a,l\right)}\right)$$

Here, $h_{j}^{\left(a,l\right)}$ denotes the embedding of atom *j* neighboring atom *i*, η(i,j) denotes the bond connecting atoms *i* and *j*, and $h_{\eta (i,j)}^{\left(b,l\right)}$ denotes the embedding of this bond. Similarly, $h_{q}^{\left(b,l\right)}$ denotes the embedding of a neighboring bond *q* of bond *p*, ξ(p,q) denotes the atom shared by bonds *p* and *q*, and $h_{\xi (p,q)}^{\left(a,l\right)}$ denotes the embedding of this atom. The matrices $W_{a}$ and $W_{b}$ are learnable linear transformations applied to atom and bond embeddings, respectively. The vectors $c_{i}^{\left(a,l\right)}$ and $c_{p}^{\left(b,l\right)}$ represent the aggregated contextual representations of atom *i* and bond *p* in the *l*-th layer. The contextual representations are then combined with the original embeddings using a learnable coefficient ϵ, followed by an MLP and a residual connection to obtain the updated embeddings:


(4) $$h_{i}^{\left(a,l+1\right)}=h_{i}^{\left(a,l\right)}+{\text{MLP}}_{a}\left((1+\epsilon )h_{i}^{\left(a,l\right)}+c_{i}^{\left(a,l\right)}\right)$$

(5) $$h_{p}^{\left(b,l+1\right)}=h_{p}^{\left(b,l\right)}+{\text{MLP}}_{b}\left((1+\epsilon )h_{p}^{\left(b,l\right)}+c_{p}^{\left(b,l\right)}\right)$$

After *L* layers of bipartite message passing, we obtain the final atom embeddings $h_{i}^{\left(a,L\right)}$. A global attention-based readout (Xiong *et al.* 2020) is then applied to derive the molecule-level representation:


(6) $$H_{b}=\text{GlobalAttention}\left({\left\{h_{i}^{\left(a,L\right)}\right\}}_{i\in V_{a}}\right),$$

where $H_{b}$ denotes the molecular representation produced by the Bipartite Graph Encoder.

#### 2.2.2 Motif transformer

To construct a comprehensive and diverse motif set for each molecule, we employ three extraction strategies—functional group matching, BRICS fragmentation, and Bemis–Murcko scaffold decomposition—and merge the extracted motifs with exact duplicates removed to form the molecule-specific motif set (see Section 3.1, available as supplementary data at *Bioinformatics* online for detailed motif definitions and extraction procedures). Non-identical overlapping or nested motifs are retained as independent motif tokens, allowing a single atom or bond to participate in multiple motifs. These retained motifs are then used as the input tokens for the Motif Transformer. Dataset-wise motif vocabulary statistics and the analysis of overlapping and nested motif settings are provided in Sections 3.2 and 3.3, available as supplementary data at *Bioinformatics* online, respectively. To assess the impact of different motif decomposition strategies, we further examined model-performance sensitivity and prediction consistency across different motif-decomposition variants in Section 4, available as supplementary data at *Bioinformatics* online.

The embedding of each motif is derived by jointly encoding fine-grained atomic contexts and higher-order interactions among motifs within a molecule. For motif *k*, its initial embedding $x_{k}^{m}$ is obtained by concatenating the sum of the initial features of the atoms and bonds it contains. Then, to enable each motif to draw contextual information from the atom embeddings learned by the Bipartite Graph Encoder, we introduce a cross-granularity communication module implemented using cross-attention and residual connections. The computation proceeds as follows:


(7) $$Q_{k}^{m}=W_{q}x_{k}^{m},\;V_{i}^{m}=W_{v}h_{i}^{\left(a,L\right)},$$

(8) $$\gamma_{ki}=\text{softmax}\left(\text{LeakyReLU}\left(z^{⊤}[Q_{k}^{m}\|V_{i}^{m}]\right)\right),$$

(9) $$h_{k}^{m}=x_{k}^{m}+\sum_{i}\gamma_{ki}V_{i}^{m}$$

where $Q_{k}^{m}$ and $V_{i}^{m}$ denote the query vector of motif *k* and the value vector of atom *i*, respectively, obtained via learnable linear projections $W_{q}$ and $W_{v}$. Here, the query vector represents the retrieval intent, while the value vectors encode the information to be aggregated in the attention mechanism. Attention weights are computed from the query–value compatibility and used to obtain a weighted sum of the value vectors. The attention coefficient $\gamma_{ki}$ measures the importance of atom *i* to motif *k*, where $z^{⊤}$ is a learnable weight vector that maps the concatenation of $Q_{k}^{m}$ and $V_{i}^{m}$ to a scalar attention score. The resulting vector $h_{k}^{m}$ denotes the atom-aware motif embedding. We further introduce a learnable CLS token to aggregate information from all motifs, which together with the atom-aware motif embeddings form the input token set:


(10) $$S=\{h_{\text{CLS}},h_{1}^{m},\ldots ,h_{K}^{m}\}$$

where *K* is the number of motifs in a molecule. This token set is fed into a *T*-layer Transformer encoder (Vaswani *et al.* 2017), where each layer consists of multi-head self-attention and position-wise feed-forward sublayers with residual connections and layer normalization. By leveraging the self-attention mechanism of the Transformer, our model effectively captures global interactions among motifs. After passing through the Transformer encoder, the output corresponding to the CLS token is taken as the motif-granularity molecular representation, denoted as $H_{m}$.

#### 2.2.3 Global Attribute Encoder

To incorporate global attribute information into the molecular representation, we introduce a Global Attribute Encoder. Specifically, molecular descriptors $d_{\text{mol}}$ extracted using RDKit (see Section 2, available as supplementary data at *Bioinformatics* online for a description of the molecular descriptors) are first standardized using the mean and standard deviation estimated from the training set, with the same transformation subsequently applied to the validation and test sets, and then fed into an MLP to produce an attribute-based query vector, which is used to draw contextual information from the atom embeddings learned by the Bipartite Graph Encoder via a cross-granularity communication module. This computation follows the same cross-granularity communication process defined in Equations (7)–(9). This process yields a global attribute–aware molecular representation, denoted as $H_{d}$.

#### 2.2.4 Fusion and prediction

To obtain a unified molecular representation that integrates complementary chemical semantics across different structural granularities, we adaptively fuse the representations $H_{b}$, $H_{m}$, and $H_{d}$ from three granularity-specific encoders using an attention mechanism. These representations are first summed and projected into a single query vector via a learnable linear transformation. In parallel, all three representations are mapped through a shared linear transformation to obtain their corresponding value vectors, denoted as $H_{v,1}$, $H_{v,2}$, and $H_{v,3}$. The final fused molecular representation is computed as


(11) $$H_{\text{fused}}=\sum_{n=1}^{3}\beta_{n}\cdot H_{v,n},$$

where $\beta_{n}$ denotes the attention weight assigned to the *n*-th representation, computed in the same manner as in Equation (8). The resulting fused representation $H_{\text{fused}}$ is subsequently fed into an MLP-based classifier or regressor to generate task-specific predictions.

The model is trained by minimizing a composite objective that consists of a supervised prediction loss and a cross-granularity alignment loss:


(12) $$L_{\text{total}}=L_{\text{pred}}+\alpha L_{\text{align}},$$

where $L_{\text{pred}}$ denotes the supervised prediction loss (binary cross-entropy for classification tasks and mean squared error (MSE) for regression tasks), and α is a hyperparameter controlling the contribution of the alignment loss. The term $L_{\text{align}}$ is a cross-granularity alignment loss designed to encourage semantic consistency between the atom–bond and motif views, and is defined as


(13) $$L_{\text{align}}=-\frac{1}{2N}\sum_{i=1}^{2N}\;\text{log}\;\frac{\;\text{exp}\;\left(\text{sim}(f_{i},f_{p(i)})/\tau \right)}{\sum_{j=1,\;j\neq i}^{2N}\;\text{exp}\;\left(\text{sim}(f_{i},f_{j})/\tau \right)},$$

where *N* is the batch size, $f_{i}$ denotes an anchor feature obtained by projecting either the atom–bond representation or the motif representation through a linear head, and $f_{p(i)}$ denotes its positive counterpart, i.e. the representation of the same molecule from the other view. Here, $\text{sim}(f_{i},f_{j})=f_{i}^{⊤}f_{j}$ denotes cosine similarity after $\ell_{2}$ normalization, and τ is a temperature hyperparameter. The analysis for α and τ can be found in Section 5, available as supplementary data at *Bioinformatics* online.

The total loss $L_{\text{total}}$ is optimized using Adam with an initial learning rate of $1\times 10^{-3}$ and a batch size of 64. Early stopping is applied based on validation performance with a patience of 50 epochs. The model is implemented in PyTorch, and all experiments are conducted on a workstation equipped with an NVIDIA RTX 4090D GPU (24 GB memory).

## 3 Results

We compare HMG-MRL with eight representative baseline methods, which can be grouped into three categories according to the types of molecular information they utilize: (i) methods that exploit only atom- and bond-level information, including AttentiveFP (Xiong *et al.* 2020), DMPNN (Yang *et al.* 2019), CMPNN (Song *et al.* 2020), ResGAT (Nguyen-Vo *et al.* 2024), as well as Transformer-based approaches such as CoMPT (Chen *et al.* 2021) and IMGT-MPN (Zhao *et al.* 2025b); (ii) descriptor-augmented methods that combine graph-based representations with traditional molecular descriptors or fingerprints, such as FP-GNN (Cai *et al.* 2022); and (iii) substructure-enhanced methods that incorporate information derived from molecular substructures, represented by HimGNN (Han *et al.* 2023). To ensure a consistent comparison, AttentiveFP, DMPNN, CMPNN, CoMPT, FP-GNN, HimGNN, and IMGT-MPN were reproduced on the same five scaffold-based splits used throughout this study. Model performance was evaluated using ROC-AUC for classification tasks and RMSE for regression tasks. For multi-task classification datasets, including ClinTox, SIDER, and Tox21, task-wise macro-averaged ROC-AUC was used. For each reproduced baseline, we followed the feature configurations and hyperparameter settings recommended in the corresponding paper or official implementation. As the source code of ResGAT was not publicly available, its results were taken from the original publication. Detailed descriptions of the baseline methods are provided in Section 6, available as supplementary data at *Bioinformatics* online. In addition, a detailed computational cost analysis, including parameter counts, peak GPU memory usage, and per-epoch training time, is provided in Section 7, available as supplementary data at *Bioinformatics* online for HMG-MRL and representative baselines, showing that HMG-MRL maintains a moderate computational cost despite its three-encoder architecture.

### 3.1 Comparison with baseline methods

Tables 1 and 2 summarize the performance of HMG-MRL and baseline methods on classification and regression tasks, respectively. Across the six classification benchmarks, HMG-MRL achieves the best average ROC-AUC on all datasets, with relatively larger mean improvements on BACE, BBBP, and ClinTox. As these three tasks are closely related to the recognition of property-relevant pharmacophores and toxicophores, the strong classification performance suggests that the Motif Transformer contributes to effective motif-level modeling of functional substructures.

**Table 1:** Predictive results of HMG-MRL and baseline methods on six classification datasets, evaluated using ROC-AUC (higher is better).^a^

Models	BACE	BBBP	SIDER	ClinTox	Tox21	HIV
AttentiveFP	0.872±0.047	0.914±0.023	0.612±0.032	0.928±0.016	0.801±0.018	0.776±0.025
DMPNN	0.863±0.054	0.925±0.024	0.633±0.016	0.906±0.042	0.815±0.011	0.782±0.010
CMPNN	0.878±0.045	0.915±0.037	0.623±0.031	0.921±0.037	0.814±0.019	0.801±0.026
CoMPT	0.855±0.047	0.930±0.027	0.627±0.018	0.929 ± 0.018	0.797±0.026	0.794±0.021
FP-GNN	0.853±0.033	0.909±0.030	0.606±0.025	0.802±0.044	0.799±0.036	0.808±0.015
HimGNN	0.861±0.053	0.931 ± 0.025	0.640 ± 0.020	0.910±0.023	0.809±0.023	0.805±0.017
ResGAT	0.823±0.030	0.876±0.040	0.600±0.060	0.866±0.010	0.827 ± 0.060	0.757±0.060
IMGT-MPN	0.884 ± 0.030	0.909±0.038	0.637±0.032	0.914±0.043	0.773±0.023	0.810 ± 0.025
**HMG-MRL**	**0.910** ± **0.025**	**0.953** ± **0.011**	**0.653** ± **0.009**	**0.947** ± **0.005**	**0.834** ± **0.010**	**0.819** ± **0.010**

a The best score in each column is highlighted in bold, and the second-best is underlined.

**Table 2:** Predictive results of HMG-MRL and baseline methods on three regression datasets, evaluated using RMSE (lower is better).^a^

Models	ESOL	FreeSolv	Lipophilicity
AttentiveFP	0.783±0.082	2.114±0.596	0.648±0.042
DMPNN	0.950±0.054	2.014±0.484	0.658±0.026
CMPNN	0.776 ± 0.090	1.929±0.643	0.631 ± 0.024
CoMPT	0.778±0.075	1.940±0.815	**0.603** ± **0.025**
FP-GNN	1.296±0.108	2.332±0.542	0.672±0.023
HimGNN	1.023±0.194	1.760±0.669	0.636±0.026
ResGAT	1.562±0.120	1.643 ± 0.220	0.717±0.040
IMGT-MPN	1.403±0.457	2.323±0.942	0.696±0.015
**HMG-MRL**	**0.690** ± **0.056**	**1.541** ± **0.559**	0.652±0.018

a The best score in each column is highlighted in bold, and the second-best is underlined.

For regression tasks, HMG-MRL achieves the best results on ESOL and FreeSolv, with an average RMSE reduction of approximately 8.6% compared with the strongest baselines on these two datasets. Since ESOL and FreeSolv are closely associated with global physicochemical characteristics such as aqueous solubility and hydration free energy, these improvements indicate the contribution of the Global Attribute Encoder and the cross-granularity communication module in incorporating descriptor-based physicochemical priors into molecular representations. On the Lipophilicity dataset, HMG-MRL performs slightly worse than the best-performing baseline. A possible explanation is that the larger and more diverse molecular distribution of Lipophilicity may diminish the relative benefit of descriptor-based global physicochemical priors. To examine this explanation, we additionally analyzed molecular structural diversity and conducted a molecular-descriptor ablation study on the three physicochemical property datasets, namely ESOL, FreeSolv, and Lipophilicity. The results indicate that Lipophilicity has a larger and more structurally diverse molecular distribution, while the relative contribution of descriptor-based global physicochemical priors is less pronounced on this dataset than on ESOL and FreeSolv. These findings help explain the slightly inferior performance of HMG-MRL on Lipophilicity. Detailed results are provided in Section 8, available as supplementary data at *Bioinformatics* online.

Moreover, one-sided paired t-tests were conducted across the five independent runs to further evaluate the statistical reliability of the performance comparisons. Most pairwise comparisons are statistically significant, whereas the few non-significant comparisons involving ClinTox, SIDER, and HIV are interpreted as numerical improvements in mean performance rather than statistically significant gains. For Lipophilicity, no superiority is claimed because HMG-MRL does not achieve the best mean RMSE. The complete statistical analysis, including *P* values, Cohen’s $d_{z}$ effect sizes, and one-sided 95% lower confidence bounds, is provided in Section 9, available as supplementary data at *Bioinformatics* online.

### 3.2 Ablation study

To assess the contributions of key components in HMG-MRL, we conduct an ablation study by evaluating six model variants: (i) w/o Bipartite Graph Encoder, which discards the atom–bond Bipartite Graph Encoder; (ii) w/o Motif Transformer, which excludes the Motif Transformer module; (iii) w/o Molecular Descriptor, which removes global molecular descriptors; (iv) w/o CGComm Module, which eliminates the cross-granularity communication module; (v) w/o Alignment Loss, which removes the cross-granularity alignment term $L_{\text{align}}$; and (vi) w/o CGComm Module and Alignment Loss, which jointly removes the cross-granularity communication module and the cross-granularity alignment term $L_{\text{align}}$, while retaining the three granularity-specific encoders.

Table 3 summarizes the ablation results on classification (BACE, BBBP) and regression (ESOL, FreeSolv) tasks. Removing the entire Bipartite Graph Encoder module (w/o Bipartite Graph Encoder) degrades performance across all tasks, indicating that jointly modeling atom–atom, atom–bond, and bond–bond interactions is crucial for learning expressive local representations. Excluding the Motif Transformer (w/o Motif Transformer) leads to clear performance degradation on both classification and regression benchmarks, reflecting the role of motif-level global interaction modeling. In addition, removing molecular descriptors (w/o Molecular Descriptor) leads to the largest performance decline on regression tasks, highlighting the importance of explicitly incorporating descriptor-based molecular attributes into the model. Removing the cross-granularity communication module (w/o CGComm Module) consistently weakens model performance, indicating the benefit of effective information exchange across different structural granularities. Eliminating the alignment loss (w/o Alignment Loss) consistently degrades performance across all four datasets, indicating that the alignment objective provides additional supervision and helps improve predictive performance. Removing both the CGComm module and the Alignment Loss (w/o CGComm Module and Alignment Loss) also leads to performance degradation across all four datasets, further supporting the contribution of these two mechanisms to the complete framework. Additional ablation results on the remaining five benchmarks are provided in Section 10, available as supplementary data at *Bioinformatics* online, and the results are consistent with those in Table 3: HMG-MRL achieves the best performance, whereas removing individual components or both the CGComm module and the Alignment Loss leads to performance degradation. To move beyond descriptive comparisons and further quantify the relative contribution of each component, we performed a quantitative component-attribution analysis based on the relative performance degradation caused by removing each module. Detailed results are provided in Section 11, available as supplementary data at *Bioinformatics* online.

**Table 3:** Ablation study results for variants of HMG-MRL.^a^

	ROC-AUC ↑		RMSE ↓	
Models	BACE	BBBP	ESOL	FreeSolv
w/o Bipartite Graph Encoder	0.890±0.018	0.938±0.012	0.751±0.086	1.664±0.720
w/o Motif Transformer	0.894±0.034	0.942±0.017	0.803±0.118	1.691±0.566
w/o Molecular Descriptor	0.897±0.028	0.945±0.015	0.845±0.132	1.785±0.618
w/o CGComm Module	0.899±0.016	0.943±0.012	0.731±0.109	1.646±0.603
w/o Alignment Loss	0.900±0.031	0.932±0.023	0.779±0.088	1.674±0.709
w/o CGComm Module and Alignment Loss	0.896±0.035	0.928±0.027	0.774±0.094	1.682±0.657
**HMG-MRL**	**0.910** ± **0.025**	**0.953** ± **0.011**	**0.690** ± **0.056**	**1.541** ± **0.559**

a The best score in each column is in bold.

Moreover, to assess whether any single molecular granularity is sufficient and whether the improvement of HMG-MRL merely arises from increased model capacity, we constructed three single-granularity variants and a parameter-matched variant. All these variants perform worse than the full HMG-MRL model, suggesting that the performance gain mainly arises from hierarchical multi-granularity integration rather than model capacity alone. Detailed results are provided in Section 12, available as supplementary data at *Bioinformatics* online. Overall, these results demonstrate that the performance gains of HMG-MRL arise from the systematic and synergistic integration of multi-granularity chemical knowledge.

### 3.3 Visualization analysis

**Figure 2:**
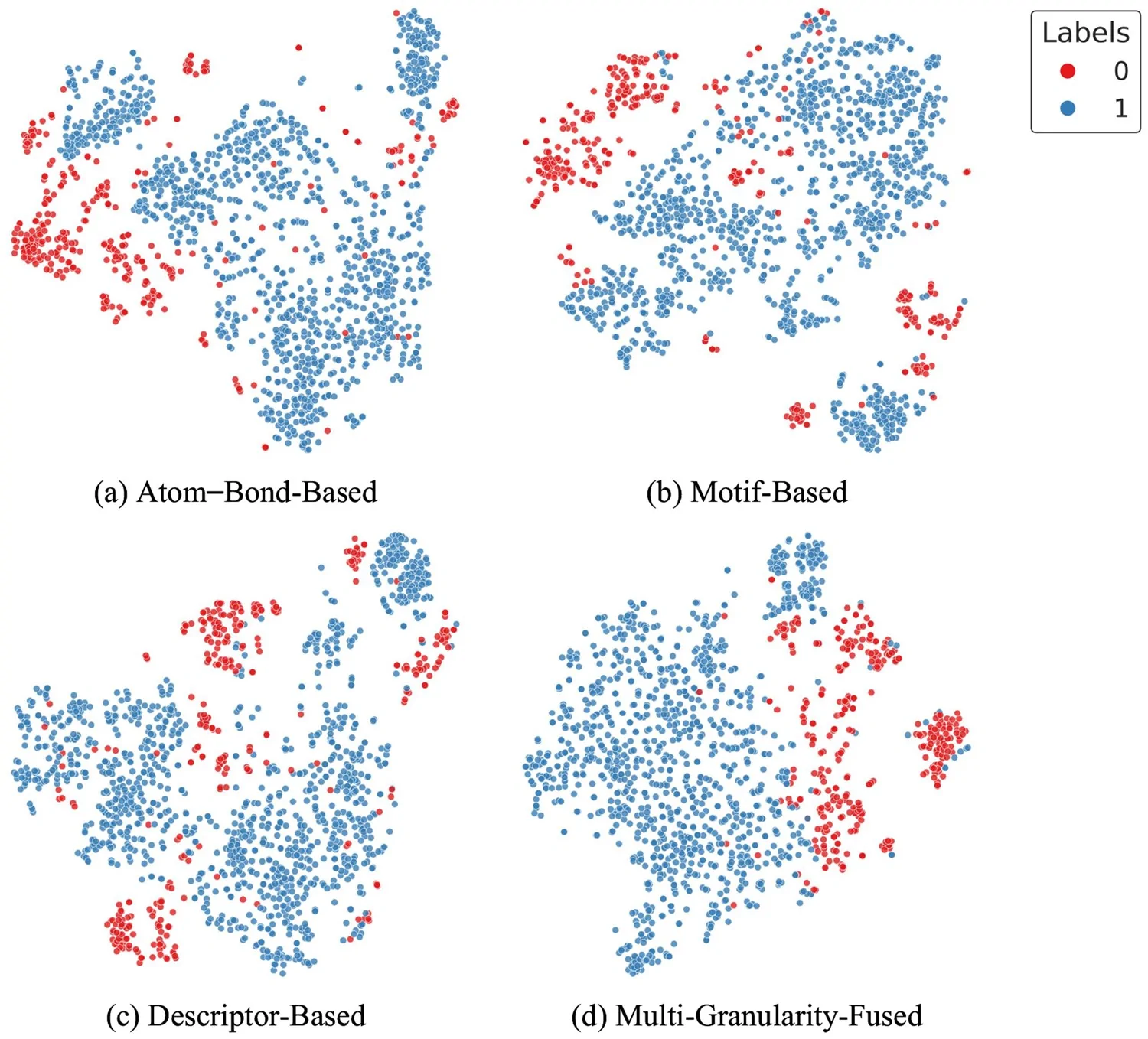
t-SNE visualizations of molecular representations on the BBBP dataset. (a) Atom–bond-based representations obtained from the Bipartite Graph Encoder. (b) Motif-based representations learned by the Motif Transformer. (c) Descriptor-based representations obtained from the Global Attribute Encoder. (d) Multi-Granularity-Fused molecular representations produced by HMG-MRL.

To provide an intuitive understanding of how multi-granularity representations contribute to molecular discrimination, we visualize the learned molecular representations on the BBBP binary classification dataset using t-SNE. Specifically, as shown in Fig. 2, molecular representations at various granularities, as well as the representations obtained via multi-granularity fusion, are projected into a two-dimensional space, with molecules colored according to their respective classes. We observe that each granularity-specific representation exhibits a certain degree of class separability (Fig. 2a–c). In contrast, the representations obtained from multi-granularity fusion show more pronounced class separation, with molecules from the two categories forming clearly defined and compact clusters (Fig. 2d). These results suggest that the multi-granularity information fusion strategy in HMG-MRL enables the model to capture richer chemical semantics, thereby enhancing its ability to discriminate between molecular classes.

Moreover, we observe that the non-penetrating compounds (label 0) in the fused representation space form several isolated regions. To examine whether these regions reflect chemically meaningful patterns related to BBB exclusion, we performed a DBSCAN-based clustering analysis and profiled the major non-penetrating regions using BBB-permeability-related physicochemical descriptors calculated by RDKit, including molecular weight, MolLogP, topological polar surface area, hydrogen bond donor count, and hydrogen bond acceptor count. Detailed cluster-level physicochemical profiles are provided in Section 13, available as supplementary data at *Bioinformatics* online and Table 19, available as supplementary data at *Bioinformatics* online. The profiling results indicate that these regions exhibit distinct physicochemical patterns associated with BBB exclusion, suggesting that the fused representation learned by HMG-MRL captures chemically meaningful patterns relevant to BBB permeability.

### 3.4 Case study

**Figure 3:**
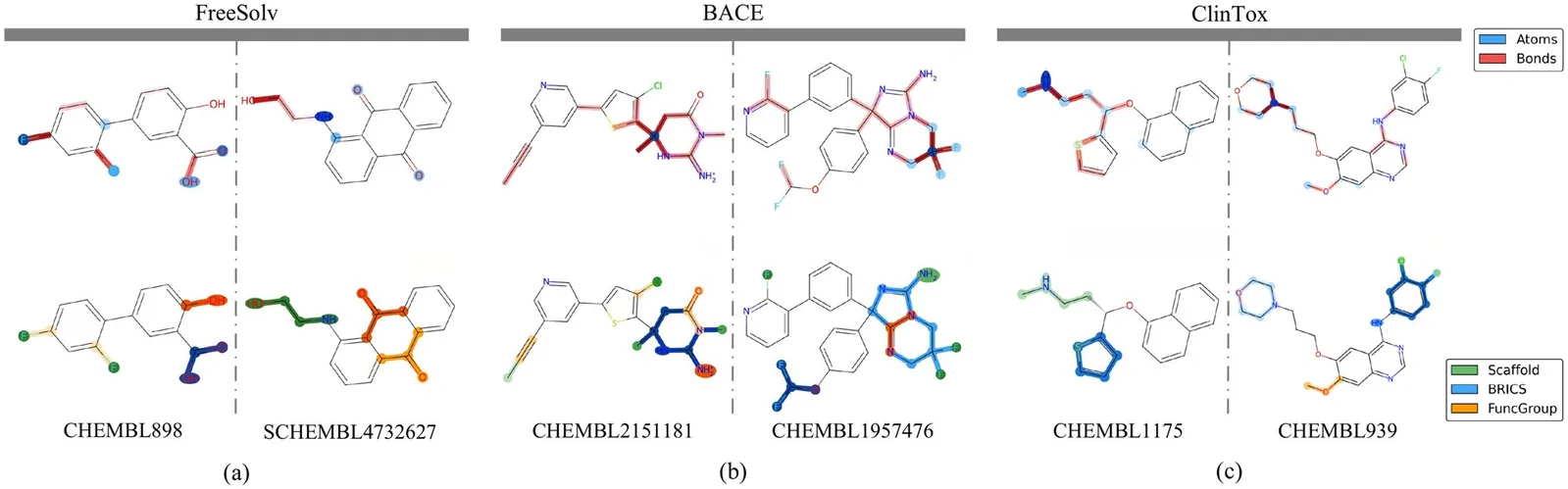
Atom–bond and motif attention visualizations of HMG-MRL. (a) CHEMBL898 and SCHEMBL4732627 from FreeSolv. (b) CHEMBL2151181 and CHEMBL1957476 from BACE. (c) CHEMBL1175 and CHEMBL939 from ClinTox. For each molecule, the upper and lower visualizations correspond to the atom–bond and motif granularities, respectively.

To examine whether HMG-MRL can identify key chemical entities relevant to molecular properties, we visualized attention weights at both the atom–bond and motif granularities for representative molecules from FreeSolv, BACE, and ClinTox (Fig. 3). For clarity, only prominent attention weights are displayed in the visualizations. The visualization results suggest that HMG-MRL can highlight property-relevant molecular regions, and these regions show spatial correspondence between the atom–bond and motif granularities.

For the FreeSolv examples (Fig. 3a), HMG-MRL assigns high attention weights to polar or locally polarized substructures at both granularities, including the carboxyl and phenolic hydroxyl groups, fluorinated aromatic regions, the hydroxyethylamino side chain, and the carbonyl-containing anthraquinone region. These highlighted substructures are associated with hydrogen-bonding interactions and local polarity, which are important physicochemical factors affecting hydration free energy. The consistent attention patterns across the atom–bond and motif views suggest that HMG-MRL can capture solvation-relevant chemical environments.

For the BACE examples, which are correctly predicted as active molecules (Fig. 3b), HMG-MRL assigns high attention weights at both granularities mainly to nitrogen-containing polar cores and adjacent aromatic or heteroaromatic fragments, including the amidine/guanidine-like cyclic core, pyridine-containing aromatic region, chlorothiophene moiety, and alkynyl substituent. These highlighted regions provide basic or positively ionizable centers, hydrogen-bonding capacity, and aromatic or hydrophobic contexts that are commonly involved in ligand recognition. The consistent attention patterns across the atom–bond and motif views support the ability of HMG-MRL to capture pharmacophore-related structural regions for BACE activity prediction.

For the ClinTox examples, which are correctly predicted as toxicity-positive molecules (Fig. 3c), HMG-MRL assigns high attention weights at both granularities to regions containing thiophene fragments, basic or protonatable side chains, and NH-linked halogenated aromatic patterns. These highlighted regions correspond to toxicity-relevant chemical contexts, including potential metabolic bioactivation of thiophenes, protonatable amine- or morpholine-containing moieties, and anilino-like aromatic patterns. The consistent attention patterns across the atom–bond and motif views suggest that HMG-MRL can capture structural alerts and toxicity-relevant molecular contexts for ClinTox toxicity prediction.

In addition, the motif visualizations across different datasets show that property-relevant motifs can arise from different substructure extraction strategies, including functional group matching, BRICS fragmentation, and Bemis–Murcko scaffolds, reflecting the complementarity of these strategies in capturing molecular patterns associated with different properties. To further examine whether these attention-highlighted components are relevant to prediction, we performed an attention-guided masking analysis on ClinTox, BACE, and FreeSolv. As detailed in Section 14, available as supplementary data at *Bioinformatics* online and Tables 20 and 21, available as supplementary data at *Bioinformatics* online, masking high-attention atoms or motifs led to greater performance degradation than random or low-attention masking. These results provide further evidence that the atom- and motif-level attention patterns learned by HMG-MRL are associated with molecular components important for prediction.

## 4 Conclusion

In this study, we propose HMG-MRL, a unified framework for hierarchical multi-granularity molecular representation learning, which integrates chemical knowledge across three granularities: fine-grained atom–bond information, medium-grained functional motifs, and coarse-grained molecular attributes. Specifically, we introduce an atom–bond bipartite graph modeling approach that treats atoms and bonds as explicit learnable node types and jointly models atom–atom, atom–bond, and bond–bond local interactions within a unified propagation framework. In parallel, we incorporate multiple substructure decomposition strategies to construct a diverse motif vocabulary and design a Motif Transformer to capture global interactions among motifs. Moreover, we design a cross-granularity communication module to facilitate information exchange across granularities. HMG-MRL achieves competitive performance across all nine benchmarks and obtains the best mean performance on eight of them, with statistically supported improvements in most pairwise comparisons. Case studies further indicate that the model can reveal key molecular components across different granularities. In future work, we will explore incorporating richer three-dimensional conformational information and geometry-aware molecular modeling into the proposed framework.

## Supplementary Material

btag600_Supplementary_Data
