# Supplementary material for: A unified framework for molecular property prediction based on hierarchical multi-granularity molecular representation learning
Source: Bioinformatics. 2026 Aug 10;42(8):btag600. doi: 10.1093/bioinformatics/btag600 (PMC13503029; doi:10.1093/bioinformatics/btag600)
Supplement: Supplementary file 1 [file btag600_supplementary_data.pdf]

Supplementary Materials for the Paper: A Unified Framework for Molecular Property Prediction based on Hierarchical Multi-Granularity Molecular Representation Learning

1 Details of Molecular Datasets

In our study, we evaluate the proposed model on nine widely used benchmark datasets derived from MoleculeNet (Wu et al., 2017), covering both molecular property classification and regression tasks. The classification benchmarks include BACE, BBBP, SIDER, ClinTox, HIV, and Tox21, while ESOL, FreeSolv, and Lipophilicity are used for regression. Collectively, these datasets span a broad range of bioactivity, physiological, toxicity, and physicochemical endpoints, enabling a comprehensive assessment of the proposed framework. Summary statistics for all datasets are provided in Table S1. Detailed descriptions of each dataset are presented below:

- **BACE**. The BACE dataset reports both quantitative (IC_{50}) and qualitative (binary) binding activities for a series of inhibitors targeting human β-secretase 1 (BACE-1). These measurements are derived from experimentally determined results published in the scientific literature over the past decade, with some compounds additionally accompanied by detailed crystal structure information. In total, the dataset contains 1,513 compounds along with their 2D structures and binary labels, and is formulated as a classification task.
- **HIV**. The HIV dataset was introduced by the Drug Therapeutics Program (DTP) as part of its AIDS antiviral screening initiative, which evaluated more than 40,000 compounds for their ability to inhibit HIV replication. The screening results were processed into a binary classification task consisting of inactive and active labels.
- **BBBP**. The Blood–Brain Barrier Penetration (BBBP) dataset originates from a study on modeling and predicting barrier permeability. As the membrane structure separating circulating blood from the extracellular fluid of the brain, the blood–brain barrier blocks most drugs, hormones, and neurotransmitters. Consequently, the ability to penetrate this barrier has long been a major challenge in the development of central nervous system–targeted therapeutics. The dataset provides binary permeability labels for over 2,000 chemical compounds.
- **ClinTox**. The ClinTox dataset is used to compare FDA-approved drugs with those that failed in clinical trials due to toxicity. It contains two classification tasks for 1,478 drug compounds: (1) clinical trial toxicity (toxic vs. non-toxic), and (2) FDA approval status.
- **Tox21**. The Tox21 dataset contains toxicity measurements for 7,831 chemical compounds across 12 different biological targets, including various nuclear receptors and stress response pathways.
- **SIDER**. The SIDER dataset partitions drug side effects into 27 system organ classes based on the MedDRA classification standard and includes measurement results for 1,427 approved drugs.
- **ESOL**. ESOL is a small dataset containing water solubility measurements for 1,128 chemical compounds. It is commonly used to train models that predict solubility directly from chemical structures encoded as Simplified Molecular-Input Line-Entry System (SMILES) strings.
- **FreeSolv**. The FreeSolv database provides experimental hydration free energies and computed hydration free energies for small molecules in water.

- **Lipophilicity**. The Lipophilicity dataset is curated from the ChEMBL database and provides experimentally measured octanol–water distribution coefficients (log D) at pH 7.4 for 4,200 chemical compounds. Lipophilicity is an important molecular property that influences membrane permeability and solubility.

Table S1: Detailed statistical information of datasets.

Dataset	Category	Task type	#Tasks	#Molecules	Metric
BACE	Bioactivity	Classification	1	1,513	ROC-AUC
HIV	Bioactivity	Classification	1	41,127	ROC-AUC
BBBP	Physiology	Classification	1	2,039	ROC-AUC
ClinTox	Toxicity	Classification	2	1,478	ROC-AUC
Tox21	Toxicity	Classification	12	7,831	ROC-AUC
SIDER	Physiology	Classification	27	1,427	ROC-AUC
ESOL	Physicochemical	Regression	1	1,128	RMSE
FreeSolv	Physicochemical	Regression	1	642	RMSE
Lipophilicity	Physicochemical	Regression	1	4,200	RMSE

2 Initial Features of Atom and Bond Nodes and Molecular Descriptors

We use RDKit (Bento et al., 2020) to extract atom and bond features as the initial features for the two types of nodes in the bipartite graph. Detailed descriptions of feature extraction are provided in Supplementary Tables S2 and S3. For the bond-length feature, three-dimensional conformers were generated from the input SMILES using RDKit. Explicit hydrogens were first added, multiple candidate conformers were generated using ETKDGv3, and the conformers were optimized with the MMFF force field. The conformer with the lowest MMFF energy was selected, and the Euclidean distance between the two bonded atoms was calculated from the resulting three-dimensional coordinates. The scalar bond length was then expanded into a 23-dimensional representation using radial basis functions. Similarly, we extract molecular descriptors (Yang et al., 2019) using RDKit, which include 200 physicochemical and structural properties, to provide prior knowledge about the global attributes of each molecule.

Table S2: Atom features used in our model.

atom feature	size	description
atom symbol	16	[B, C, N, O, F, Si, P, S, Cl, As, Se, Br, Te, I, At, metal] (one-hot)
degree	6	number of covalent bonds [0,1,2,3,4,5] (one-hot)
formal charge	1	electrical charge (integer)
radical electrons	1	number of radical electrons (integer)
hybridization	6	[sp, sp^2, sp^3, sp^3d, sp^3d^2, other] (one-hot)
aromaticity	1	whether the atom is part of an aromatic system [0/1] (one-hot)
hydrogens	5	number of connected hydrogens [0,1,2,3,4] (one-hot)
chirality	1	whether the atom is a chiral center [0/1] (one-hot)
chirality type	2	[R, S] (one-hot)

3 Motif Definition, Vocabulary Statistics, and Overlapping/Nested Motif Analysis

3.1 Motif definition and extraction strategies

In this study, we define molecular motifs as chemically meaningful substructures that capture local functional patterns, fragment-level structural units, or scaffold-level molecular frameworks. To construct a

Table S3: Bond features used in our model.

bond feature	size	description
bond type	4	[single, double, triple, aromatic] (one-hot)
conjugation	1	whether the bond is conjugated [0/1] (one-hot)
ring	1	whether the bond is in a ring [0/1] (one-hot)
stereo	4	[StereoNone, StereoAny, StereoZ, StereoE] (one-hot)
electronegativity diff	9	Pauling electronegativity difference across the bond, reflecting bond polarity (encoded with radial basis functions).
bond length	23	Euclidean distance between bonded atoms calculated from the RDKit-generated three-dimensional conformer and encoded with radial basis functions.

comprehensive and diverse motif set for each molecule, we employ three rule-based substructure extraction strategies and merge the resulting motifs into a molecule-specific motif set after removing exact duplicates. Non-identical overlapping or nested motifs are retained as independent motif tokens. The three extraction strategies are described as follows:

- Functional Groups: Specific atomic groups that significantly influence the physicochemical properties and characteristic chemical reactions of molecules.
- BRICS Fragments (Degen et al., 2008): Based on retrosynthetic analysis, molecules are decomposed into a series of substructures by breaking chemically tractable bonds that are likely to form or break during chemical reactions.
- Bemis–Murcko Scaffolds and Side Chains (Wu et al., 2023): Core topological frameworks (Bemis–Murcko scaffolds) and their associated peripheral modifications (side chains) extracted from complex molecular structures.

Table S4: Dataset-wise motif statistics.

Dataset	#Molecules	#Unique BRICS Motifs	#Unique Bemis–Murcko Scaffold Motifs	#Unique SMARTS-Matched Functional Motifs	#Unique Motifs in the Merged Set	Avg. #Motifs per Molecule
BACE	1,513	573	982	130	1,597	18.38
BBBP	2,039	1,313	1,514	255	2,803	13.65
SIDER	1,427	1,236	1,412	277	2,638	20.88
ClinTox	1,478	1,184	1,306	309	2,527	15.38
Tox21	7,831	2,704	3,370	538	6,013	10.33
HIV	41,127	15,299	20,620	945	34,394	13.46
ESOL	1,128	340	364	173	771	6.82
FreeSolv	642	115	112	108	273	4.32
Lipophilicity	4,200	1,640	2,779	205	4,395	14.49

3.2 Dataset-wise motif vocabulary statistics

To further characterize the motif sets used in HMG-MRL, we report dataset-wise motif vocabulary statistics across the nine benchmark datasets. For each dataset, we summarize the number of molecules, the number of unique motifs generated by each extraction strategy, the number of unique motifs in the merged motif set, and the average number of motif tokens per molecule. The results are shown in Supplementary Table S4.

3.3 Analysis of Overlapping and Nested Motif Settings

In HMG-MRL, motif candidates are generated by functional group matching, BRICS fragmentation, and Bemis–Murcko scaffold decomposition. After exact duplicate motifs are removed, non-identical motifs

are retained as independent motif tokens, even if they partially overlap or one motif is nested within another. Specifically, two motifs are considered nested if the atom/bond set of one motif fully contains, or is fully contained by, that of another motif. Two motifs are considered overlapping if they share at least one atom or bond but neither fully contains the other. This setting retains overlapping or nested motifs because they may capture complementary chemical information at different structural resolutions. To examine whether retaining these overlapping or nested motifs affects learning stability, we compared HMG-MRL with two stricter motif-filtering settings on BACE (classification) and ESOL (regression):

- Filtered non-overlapping motifs: retained motifs do not share atoms or bonds with each other.
- Filtered de-nested motifs: no retained motif fully contains or is fully contained by another retained motif.

As shown in Supplementary Table S5, HMG-MRL achieves the best performance on both datasets and also shows smaller standard deviations than the two filtered settings. These results show that retaining non-identical overlapping or nested motifs does not impair learning stability, and that such motifs provide complementary structural information for molecular property prediction.

Table S5: Performance comparison of different motif filtering settings on BACE and ESOL. The best score in each column is highlighted in bold.

Motif Filtering Setting	BACE (ROC-AUC ↑)	ESOL (RMSE ↓)
Filtered non-overlapping motifs	0.893 ± 0.036	0.775 ± 0.103
Filtered de-nested motifs	0.901 ± 0.040	0.793 ± 0.101
**HMG-MRL**	**0.910 ± 0.025**	**0.690 ± 0.056**

4 Sensitivity Analysis of Motif Decomposition Strategies

Given that the motif-level branch of HMG-MRL is built upon motif vocabularies derived from SMARTS-based functional groups, BRICS fragments, and Bemis–Murcko scaffolds, we further examined the sensitivity of model performance to different motif decomposition strategies and the consistency of the resulting predictive behavior. To examine the contribution and robustness of these strategies, we constructed six motif-strategy variants by using different motif vocabularies: BRICS fragments only, Bemis–Murcko scaffolds only, SMARTS-based functional groups only, BRICS fragments + Bemis–Murcko scaffolds, BRICS fragments + functional groups, and Bemis–Murcko scaffolds + functional groups. The full HMG-MRL model uses the integrated motif vocabulary derived from all three strategies.

We selected BACE and ESOL as representative classification and regression benchmarks, respectively. For all motif-strategy variants, only the motif decomposition strategy was changed, while the scaffold-based data splits, the remaining model architecture, training settings, and evaluation metrics were kept identical. All results were obtained from five independent scaffold-based runs and are reported as mean ± standard deviation. ROC-AUC was used for BACE, and RMSE was used for ESOL.

As shown in Supplementary Table S6, different motif vocabularies led to certain performance variations, indicating that the choice of motif decomposition strategy can affect predictive performance. Nevertheless, all motif-strategy variants retained predictive utility on both datasets. On BACE, the single- or two-strategy variants achieved ROC-AUC values ranging from 0.888 to 0.900, while the full HMG-MRL model achieved the best mean ROC-AUC of 0.910. On ESOL, the performance differences among motif strategies were more pronounced, especially for BRICS-only and scaffold-only settings. However, the full HMG-MRL model still achieved the lowest mean RMSE of 0.690. These results suggest that integrating BRICS fragments, Bemis–Murcko scaffolds, and functional groups provides complementary structural information and leads to the best overall performance on both representative tasks.

To further examine whether different motif decomposition strategies lead to consistent predictive behavior, we calculated the Spearman correlation between the test-set predictions of each motif-strategy variant and those of the full HMG-MRL model. For BACE, the predicted classification probabilities were used for correlation analysis, while for ESOL, the predicted regression values were used. The correlations were computed separately for each scaffold run and then summarized as mean ± standard deviation across five runs.

As shown in Supplementary Table S7, all motif-strategy variants exhibited high prediction correlations with the full HMG-MRL model. The Spearman correlation ranged from 0.894 to 0.912 on BACE and from 0.956 to 0.982 on ESOL. These results indicate that although different motif vocabularies affect predictive performance to some extent, they lead to broadly consistent predictive trends. Overall, HMG-MRL is not overly dependent on any single motif decomposition strategy; instead, the integrated motif vocabulary provides complementary structural information while maintaining stable predictive behavior.

Table S6: Performance sensitivity analysis under different motif decomposition strategy settings. The best score in each column is highlighted in bold.

Motif strategy setting	BACE (ROC-AUC ↑)	ESOL (RMSE ↓)
BRICS only	0.896 ± 0.036	0.933 ± 0.380
Scaffold only	0.889 ± 0.033	0.908 ± 0.280
Functional group only	0.888 ± 0.037	0.741 ± 0.077
BRICS + Scaffold	0.890 ± 0.022	0.715 ± 0.081
BRICS + Functional group	0.900 ± 0.034	0.768 ± 0.067
Scaffold + Functional group	0.893 ± 0.028	0.758 ± 0.055
**HMG-MRL**	**0.910 ± 0.025**	**0.690 ± 0.056**

Table S7: Prediction consistency with HMG-MRL under different motif decomposition strategy settings.

Motif strategy setting	BACE (Spearman Corr.)	ESOL (Spearman Corr.)
BRICS only	0.909 ± 0.025	0.965 ± 0.016
Scaffold only	0.911 ± 0.033	0.956 ± 0.032
Functional group only	0.905 ± 0.024	0.976 ± 0.011
BRICS + Scaffold	0.894 ± 0.032	0.979 ± 0.011
BRICS + Functional group	0.912 ± 0.029	0.982 ± 0.009
Scaffold + Functional group	0.908 ± 0.021	0.976 ± 0.006

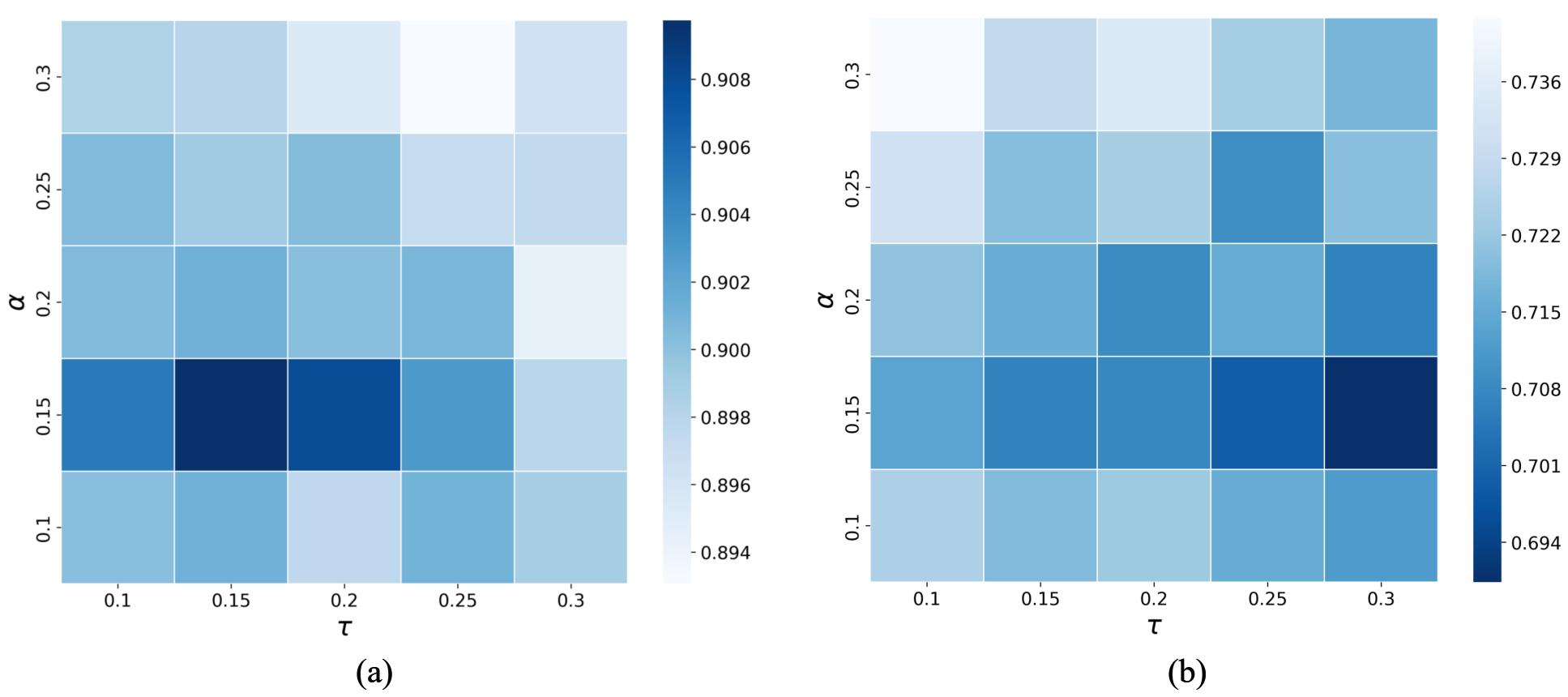

Figure S1: Hyperparameter analysis of α and τ on BACE (a) and ESOL (b).

5 Hyperparameter Analysis

We explored two important hyperparameters of HMG-MRL: the coefficient α that balances the supervised prediction loss and the cross-granularity alignment loss in Eq. (12), and the temperature coefficient τ in Eq. (13). These hyperparameters were selected according to task type. Specifically, BACE was used as the representative classification dataset, and ESOL was used as the representative regression dataset. For each representative dataset, we performed a grid search over 25 combinations of α and τ, where both α and τ were selected from $\{0.10, 0.15, 0.20, 0.25, 0.30\}$. Each setting was independently run five times using different random seeds, and the mean performance was reported.

As shown in Supplementary Fig. S1(a), the combination of $\alpha = 0.15$ and $\tau = 0.15$ achieved the highest mean ROC-AUC on BACE; therefore, this setting was used for all classification datasets. As shown in Supplementary Fig. S1(b), the combination of $\alpha = 0.15$ and $\tau = 0.30$ achieved the lowest mean RMSE on ESOL; therefore, this setting was used for all regression datasets. Although the BACE results show a relatively stable plateau around $\tau = 0.15$, this value achieved the best mean ROC-AUC among the predefined candidate values. The stable performance of neighboring values further indicates that HMG-MRL is robust to the selection of τ within this range.

6 Detailed Description of Baselines

We compare HMG-MRL with eight representative baseline methods, which can be grouped into three categories according to the types of molecular information they utilize: (i) methods that exploit only atomic and bond information, including AttentiveFP (Xiong et al., 2020), DMPNN (Yang et al., 2019), CMPNN (Song et al., 2020), and ResGAT (Nguyen-Vo et al., 2024); (ii) descriptor-augmented approaches that combine graph-based representations with traditional molecular descriptors or fingerprints, such as FP-GNN (Cai et al., 2022); and (iii) substructure-enhanced methods that incorporate information derived from molecular substructures, exemplified by HimGNN (Han et al., 2023). In addition, we also compare against two Transformer-based graph methods, CoMPT (Chen et al., 2021) and IMGT-MPN (Zhao et al., 2025), which employ self-attention mechanisms to perform message passing on molecular graphs. The detailed descriptions of these baselines are as follows:

- AttentiveFP leverages atom-level attention to weight neighboring atoms during message passing, enabling more expressive atomic representations. Stacked attentive layers and a global super-node further integrate local and nonlocal atomic information for molecular property prediction.
- DMPNN centers message passing on directed bonds, treating bonds rather than atoms as the primary carriers of structural information. This bond-focused design avoids redundant atom-level loops and yields more stable encoding of local chemical interactions. By aggregating bond-derived representations into atom and molecular embeddings, the model captures key bonding patterns that drive molecular property prediction.
- CMPNN strengthens molecular graph representation by enabling explicit communication between atoms and bonds during message passing. Unlike MPNN or DMPNN, CMPNN updates both node and edge embeddings interactively and introduces a message booster to amplify informative bond messages. This bidirectional atom–bond interaction leads to richer structural representations and improved molecular property prediction.
- ResGAT enhances molecular representation by stacking graph attention layers with residual shortcut connections, enabling stable and deeper propagation of atom-level features. Each block integrates attention-based updates with skip connections to preserve informative local atomic patterns, followed by global pooling for final molecular embeddings.

- FP-GNN fuses atom-level graph attention features with complementary molecular fingerprints to form a hybrid representation. Node embeddings from the GNN are combined with fingerprint-derived substructure information, enabling the model to capture both learned atomic interactions and predefined chemical patterns for property prediction.
- HimGNN learns molecular representations by jointly modeling atom-level and motif-level graphs. It uses Atom-MPNN to encode atomic interactions and Motif-MPNN to capture higher-order substructures, while a Transformer-based augmentation injects atom information into motif features. A contextual self-rescaling module then adaptively balances atom and motif embeddings, yielding a hierarchical representation for molecular property prediction.
- CoMPT adopts a Transformer-style architecture for molecular graphs, replacing self-attention with a node–edge communicative interaction module that jointly updates atom and bond embeddings. A topology-aware diffusion mechanism constrains attention using graph structure, enabling Transformer-level global reasoning while avoiding message explosion and improving molecular representation quality.
- IMGT-MPN strengthens a Graph Transformer by adding relative position and bond-type encodings into self-attention, allowing the model to capture molecular topology more explicitly. By alternately stacking MPNN layers with global Transformer attention, the architecture integrates local chemical interactions with long-range dependencies, yielding more expressive molecular representations.

7 Computational Cost and Practical Feasibility Analysis

To assess the practical feasibility of HMG-MRL, we compared its computational cost with representative baseline methods on one classification dataset, BBBP, and one regression dataset, ESOL. Three computational cost metrics were reported: the number of trainable parameters, peak GPU memory usage during training, and average training time per epoch. All measurements were conducted under the same hardware environment, using one NVIDIA RTX 4090D GPU with 24 GB memory and a 16-vCPU Intel Xeon Platinum 8474C CPU.

As shown in Supplementary Tables S8 and S9, HMG-MRL maintains a moderate computational cost compared with representative baselines. On the BBBP dataset, HMG-MRL contains 1.234M trainable parameters, uses 1.660 GB peak GPU memory, and requires 5.940 s per epoch on average. Its parameter count is lower than those of CMPNN, CoMPT, FP-GNN, and IMGT-MPN, its peak GPU memory usage is lower than those of CoMPT and IMGT-MPN, and its per-epoch training time is shorter than those of CoMPT, FP-GNN, and IMGT-MPN. On the ESOL dataset, HMG-MRL contains 2.153M trainable parameters, uses 1.555 GB peak GPU memory, and requires 2.110 s per epoch on average. Its per-epoch training time is shorter than those of CMPNN, FP-GNN, and IMGT-MPN. These values remain within a practical range for single-GPU training and are comparable to those of representative molecular property prediction models.

Overall, the computational cost analysis indicates that HMG-MRL achieves improved predictive performance with a manageable computational cost. Although the model integrates three encoders and the cross-granularity communication module, its parameter count, memory usage, and per-epoch training time remain practical on standard GPU hardware. ResGAT was not included in this comparison because its publicly available implementation was unavailable, preventing fair measurement under the same experimental environment.

Table S8: Computational cost comparison on the BBBP dataset.

Model	Params (M)	Peak GPU Memory (GB)	Avg. Training Time per Epoch (s)
AttentiveFP	0.808	0.647	1.445
DMPNN	0.409	0.100	0.903
CMPNN	2.023	0.423	2.651
CoMPT	2.626	4.471	7.597
FP-GNN	1.487	0.405	13.044
HimGNN	0.943	1.106	2.178
IMGT-MPN	2.334	1.921	57.503
HMG-MRL	1.234	1.660	5.940

Table S9: Computational cost comparison on the ESOL dataset.

Model	Params (M)	Peak GPU Memory (GB)	Avg. Training Time per Epoch (s)
AttentiveFP	1.145	0.375	0.523
DMPNN	0.409	0.068	0.552
CMPNN	2.023	0.222	2.267
CoMPT	2.219	2.476	1.363
FP-GNN	1.487	0.136	7.924
HimGNN	2.078	1.638	1.366
IMGT-MPN	2.334	0.583	6.881
HMG-MRL	2.153	1.555	2.110

8 Molecular Structural Diversity and Molecular-Descriptor Ablation for Physicochemical Property Datasets

To further investigate the performance difference of HMG-MRL among ESOL, FreeSolv, and Lipophilicity, we analyzed dataset scale, scaffold-based structural diversity, and the contribution of the molecular descriptor branch on these physicochemical property datasets.

First, we analyzed whether Lipophilicity exhibits a more diverse molecular distribution than ESOL and FreeSolv. Bemis–Murcko scaffolds are commonly used to characterize the structural diversity of molecular datasets; therefore, the number of unique scaffolds and the scaffold/molecule ratio can reflect the diversity and dispersion of the molecular chemical space. As shown in Supplementary Table S10, Lipophilicity contains 4,200 molecules and 2,779 unique scaffolds, with a scaffold/molecule ratio of 0.662, which is substantially higher than those of ESOL and FreeSolv. This result indicates that Lipophilicity covers a larger and more structurally dispersed molecular space, making scaffold-level generalization more challenging.

Second, we examined the relative contribution of the molecular descriptor branch across these three physicochemical property datasets. Since lipophilicity is closely related to global physicochemical properties, molecular descriptors are expected to provide useful prior knowledge for this task. However, on a dataset with larger scale and higher scaffold diversity, the relative advantage brought by such descriptor-based prior information may become less pronounced. To examine this point, we additionally evaluated the variant without molecular descriptors on Lipophilicity and compared its relative performance change with the corresponding results on ESOL and FreeSolv. As shown in Supplementary Table S11, removing molecular descriptors increases RMSE by 22.46% on ESOL and 15.83% on FreeSolv, but only by 2.91% on Lipophilicity. These results indicate that although molecular descriptors remain useful for Lipophilicity, the performance gain introduced by descriptor-based global physicochemical priors is much less pronounced on Lipophilicity than on ESOL and FreeSolv.

Taken together, these two analyses show that Lipophilicity has a larger and more structurally diverse

molecular distribution, and that the advantage of the molecular descriptor branch becomes relatively less evident on this dataset. This provides a more detailed explanation for why HMG-MRL achieves slightly inferior performance compared with the strongest baseline on Lipophilicity, while still achieving the best performance on ESOL and FreeSolv.

Table S10: Dataset-scale and scaffold-diversity analysis of ESOL, FreeSolv, and Lipophilicity.

Dataset	#Molecules	#Unique scaffolds	Scaffold/Molecule ratio
ESOL	1,128	364	0.323
FreeSolv	642	112	0.174
Lipophilicity	4,200	2,779	0.662

Table S11: Effect of removing molecular descriptors on ESOL, FreeSolv, and Lipophilicity.

Dataset	HMG-MRL	w/o Molecular Descriptor	Relative RMSE increase
ESOL	0.690±0.056	0.845±0.132	22.46%
FreeSolv	1.541±0.559	1.785±0.618	15.83%
Lipophilicity	0.652±0.018	0.671±0.040	2.91%

9 Statistical Significance Analysis

To further evaluate the statistical reliability of the performance comparisons, paired significance testing was conducted between HMG-MRL and all reproduced baseline methods with publicly available code (AttentiveFP, DMPNN, CMPNN, CoMPT, FP-GNN, HimGNN, and IMGT-MPN) using the run-level results from the same five scaffold-based splits. ResGAT was excluded from this analysis owing to the lack of a public implementation; its results were taken from the original publication rather than reproduced under the same split-wise protocol. Because the statistical question was inherently directional—namely, whether HMG-MRL outperformed each reproduced baseline—we adopted directional alternative hypotheses and, accordingly, one-sided paired t-tests. For classification tasks, the alternative hypothesis was that HMG-MRL achieved a higher ROC-AUC than the corresponding baseline method, whereas for regression tasks, it was that HMG-MRL achieved a lower RMSE. These directional hypotheses were applied uniformly across all datasets and baselines, regardless of the observed direction of the performance differences. The resulting one-sided p-values and the corresponding one-sided 95% lower confidence bounds for the mean paired performance differences are reported in Supplementary Tables S12 and S13, respectively.

As shown in Supplementary Table S12, most pairwise comparisons reach the 0.05 significance threshold, indicating statistically supported improvements in most cases. On the Lipophilicity dataset, HMG-MRL does not achieve the best mean RMSE among the compared methods, which may be related to the larger and more structurally diverse molecular distribution of this dataset, as discussed in the manuscript. The four non-significant p-values on Lipophilicity are consistent with this mean-performance comparison. Apart from Lipophilicity, only four comparisons do not reach the 0.05 significance threshold, namely ClinTox vs CMPNN, ClinTox vs IMGT-MPN, SIDER vs IMGT-MPN, and HIV vs IMGT-MPN. For these cases, HMG-MRL achieves better mean performance, but the paired tests do not support statistically significant superiority. These cases are therefore interpreted as numerical improvements rather than statistically significant gains.

In addition to p-values, Cohen's d_z effect sizes and one-sided 95% lower confidence bounds for the mean paired performance differences were reported to quantify the magnitude and uncertainty of the performance differences. For each comparison, paired split-wise performance differences were calculated across the same five scaffold-based splits. For classification tasks, the paired difference was defined as

the ROC-AUC of HMG-MRL minus that of the baseline method, whereas for regression tasks, it was defined as the RMSE of the baseline method minus that of HMG-MRL. Cohen's d_z was calculated as the mean paired difference divided by the standard deviation of the paired differences. Under these difference definitions, a positive Cohen's d_z indicates better average performance of HMG-MRL for both classification and regression tasks. The one-sided 95% lower confidence bound was obtained from the t-based confidence interval for the mean paired difference, and a lower bound above zero indicates statistical support for a directional improvement of HMG-MRL. The results are reported in Supplementary Table S13. As shown in Supplementary Table S13, most comparisons yield positive Cohen's d_z values and positive 95% lower confidence bounds, consistent with the p-value analysis. For the few non-significant comparisons, the lower confidence bounds are at or below zero, supporting our interpretation of these cases as numerical improvements rather than statistically significant gains. For Lipophilicity, the effect-size and confidence-bound results are also consistent with the mean-performance comparison.

Table S12: One-sided p-values of paired t-tests between HMG-MRL and each reproduced baseline using the same five scaffold-based splits.

Dataset	AttentiveFP	DMPNN	CMPNN	CoMPT	FP-GNN	HimGNN	IMGT-MPN
BACE	0.013	0.021	0.010	0.009	0.006	0.011	0.013
BBBP	0.014	0.009	0.016	0.016	0.017	0.024	0.016
ClinTox	0.019	0.033	0.064	0.018	0.001	0.022	0.056
SIDER	0.011	0.018	0.015	0.017	0.015	0.022	0.085
HIV	0.012	0.005	0.029	0.009	0.016	0.011	0.118
Tox21	0.020	0.007	0.013	0.015	0.022	0.017	0.001
ESOL	0.010	0.003	0.020	0.015	0.001	0.016	0.018
FreeSolv	0.014	0.017	0.006	0.037	0.023	0.014	0.017
Lipophilicity	0.558	0.039	0.982	0.968	0.018	0.951	0.004

Table S13: Cohen's d_z effect sizes and one-sided 95% lower confidence bounds for the mean paired performance differences between HMG-MRL and each reproduced baseline using the same five scaffold-based splits.

Dataset	AttentiveFP	DMPNN	CMPNN	CoMPT	FP-GNN	HimGNN	IMGT-MPN
BACE	1.533<br>$[0.014, +\infty)$	1.318<br>$[0.013, +\infty)$	1.678<br>$[0.014, +\infty)$	1.731<br>$[0.024, +\infty)$	1.953<br>$[0.029, +\infty)$	1.627<br>$[0.020, +\infty)$	1.544<br>$[0.010, +\infty)$
BBBP	1.495<br>$[0.014, +\infty)$	1.726<br>$[0.013, +\infty)$	1.459<br>$[0.013, +\infty)$	1.444<br>$[0.008, +\infty)$	1.412<br>$[0.014, +\infty)$	1.257<br>$[0.005, +\infty)$	1.438<br>$[0.015, +\infty)$
ClinTox	1.365<br>$[0.006, +\infty)$	1.121<br>$[0.006, +\infty)$	0.856<br>$[-0.003, +\infty)$	1.402<br>$[0.006, +\infty)$	3.423<br>$[0.105, +\infty)$	1.303<br>$[0.010, +\infty)$	0.906<br>$[-0.002, +\infty)$
SIDER	1.613<br>$[0.017, +\infty)$	1.387<br>$[0.006, +\infty)$	1.471<br>$[0.011, +\infty)$	1.416<br>$[0.009, +\infty)$	1.473<br>$[0.017, +\infty)$	1.298<br>$[0.003, +\infty)$	0.746<br>$[-0.005, +\infty)$
HIV	1.573<br>$[0.017, +\infty)$	2.065<br>$[0.020, +\infty)$	1.171<br>$[0.003, +\infty)$	1.730<br>$[0.011, +\infty)$	1.439<br>$[0.004, +\infty)$	1.627<br>$[0.006, +\infty)$	0.622<br>$[-0.005, +\infty)$
Tox21	1.337<br>$[0.009, +\infty)$	1.844<br>$[0.009, +\infty)$	1.544<br>$[0.008, +\infty)$	1.459<br>$[0.013, +\infty)$	1.294<br>$[0.009, +\infty)$	1.402<br>$[0.008, +\infty)$	3.584<br>$[0.045, +\infty)$
ESOL	1.655<br>$[0.039, +\infty)$	2.350<br>$[0.154, +\infty)$	1.334<br>$[0.024, +\infty)$	1.468<br>$[0.031, +\infty)$	3.552<br>$[0.443, +\infty)$	1.459<br>$[0.115, +\infty)$	1.386<br>$[0.223, +\infty)$
FreeSolv	1.497<br>$[0.208, +\infty)$	1.408<br>$[0.153, +\infty)$	1.929<br>$[0.196, +\infty)$	1.073<br>$[0.044, +\infty)$	1.273<br>$[0.198, +\infty)$	1.525<br>$[0.082, +\infty)$	1.409<br>$[0.253, +\infty)$
Lipophilicity	-0.070<br>$[-0.064, +\infty)$	1.052<br>$[0.001, +\infty)$	-1.377<br>$[-0.036, +\infty)$	-1.142<br>$[-0.091, +\infty)$	1.383<br>$[0.006, +\infty)$	-0.965<br>$[-0.033, +\infty)$	2.125<br>$[0.024, +\infty)$

Note: Each cell reports Cohen's d_z on the first line and the one-sided 95% lower confidence bound for the mean paired performance difference in brackets on the second line.

10 Ablation Results on the Remaining Five Benchmarks

In the main text, Table 3 reports the ablation results of HMG-MRL on four representative benchmarks, including two classification benchmarks (BACE and BBBP) and two physicochemical property benchmarks (ESOL and FreeSolv). To provide a more complete evaluation of the contributions of different

model components, we further report the ablation results on the remaining five benchmarks, namely HIV, ClinTox, SIDER, Tox21, and Lipophilicity, in Supplementary Table S14. The results on these remaining five benchmarks are consistent with the observations reported in the main text. The complete HMG-MRL model achieves the best performance on all five benchmarks. Removing the Bipartite Graph Encoder, the Motif Transformer, the Molecular Descriptor branch, the cross-granularity communication module, or the alignment loss, as well as jointly removing the cross-granularity communication module and the alignment loss, consistently leads to performance degradation. These results further support the contribution of the different components and their coordinated integration to the predictive performance of HMG-MRL.

Table S14: Ablation results of HMG-MRL on the remaining five benchmarks. The best score in each column is in bold.

Model	ROC-AUC ↑				RMSE ↓
	**HIV**	**ClinTox**	**SIDER**	**Tox21**	**Lipophilicity**
w/o Bipartite Graph Encoder	0.799±0.014	0.912±0.024	0.620±0.034	0.819±0.025	0.721±0.023
w/o Motif Transformer	0.792±0.027	0.924±0.019	0.637±0.025	0.823±0.022	0.675±0.018
w/o Molecular Descriptor	0.796±0.041	0.930±0.009	0.629±0.042	0.811±0.020	0.671±0.040
w/o CGComm Module	0.807±0.022	0.940±0.012	0.623±0.032	0.817±0.024	0.673±0.026
w/o Alignment Loss	0.814±0.026	0.935±0.005	0.640±0.028	0.821±0.026	0.672±0.013
w/o CGComm Module and Alignment Loss	0.803±0.032	0.929±0.015	0.627±0.039	0.815±0.032	0.681±0.038
**HMG-MRL**	**0.819±0.010**	**0.947±0.005**	**0.653±0.009**	**0.834±0.010**	**0.652±0.018**

11 Quantitative Component-Attribution Analysis

To further quantify the component-wise contributions of HMG-MRL, we performed a quantitative component-attribution analysis based on the relative performance degradation caused by removing each module. This analysis was derived from the component-removal ablation results reported in Table 3 and Supplementary Table S14, which cover all nine benchmark datasets.

For classification datasets, the contribution of each component was assessed using the relative ROC-AUC decrease after removing the corresponding module. Specifically, the relative ROC-AUC decrease was calculated as the difference between the ROC-AUC of HMG-MRL and that of the corresponding ablated variant, divided by the ROC-AUC of HMG-MRL and multiplied by 100%. For regression datasets, the relative RMSE increase was calculated as the difference between the RMSE of the corresponding ablated variant and that of HMG-MRL, divided by the RMSE of HMG-MRL and multiplied by 100%. Therefore, larger percentages indicate greater relative performance degradation after removing the corresponding component.

The results on classification datasets are shown in Supplementary Table S15. Removing the Bipartite Graph Encoder leads to the largest average relative ROC-AUC decrease, indicating that the atom–bond representation plays an important role in classification tasks. Jointly removing the CGComm module and the Alignment Loss causes the second-largest average relative ROC-AUC decrease, suggesting that cross-granularity communication and semantic-consistency alignment together provide important support for classification performance. Removing either the Molecular Descriptor branch or the Motif Transformer also causes consistent relative performance degradation, suggesting that molecular attributes and motif-granularity representations provide complementary information for classification.

The results on regression datasets are shown in Supplementary Table S16. Removing the Molecular Descriptor branch results in the largest average relative RMSE increase, especially on ESOL and FreeSolv, indicating that descriptor-based global molecular attributes are particularly useful for physicochemical property prediction. Removing either the Motif Transformer or the Bipartite Graph Encoder also increases RMSE across the regression datasets, further supporting the contribution of motif-granularity and atom–bond representations.

We further clarified the isolated and joint roles of the CGComm module and the Alignment Loss. The w/o CGComm Module variant removes the cross-granularity communication module while retaining the three granularity-specific encoders and the cross-granularity alignment loss. Therefore, its performance degradation reflects the contribution of explicit information exchange across atom–bond, motif, and molecular descriptor granularities. In contrast, the w/o Alignment Loss variant retains the CGComm module but removes the cross-granularity alignment objective, thereby isolating the contribution of semantic-consistency regularization between the atom–bond and motif views. The w/o CGComm Module and Alignment Loss variant removes both mechanisms to examine their joint effect. As shown in Supplementary Tables S15 and S16, removing either CGComm or the Alignment Loss leads to consistent relative performance degradation across classification and regression datasets, while jointly removing both mechanisms causes larger average relative degradation. These results indicate that CGComm and the alignment objective make distinct and partially complementary contributions to HMG-MRL.

Table S15: Component-attribution analysis on classification datasets based on the relative ROC-AUC decrease (%) after removing each module.

Ablation variant	BACE	BBBP	SIDER	ClinTox	Tox21	HIV	Avg. relative decrease (%)
w/o Bipartite Graph Encoder	2.20	1.57	5.05	3.70	1.80	2.44	2.79
w/o Motif Transformer	1.76	1.15	2.45	2.43	1.32	3.30	2.07
w/o Molecular Descriptor	1.43	0.84	3.68	1.80	2.76	2.81	2.22
w/o CGComm Module	1.21	1.05	4.59	0.74	2.04	1.47	1.85
w/o Alignment Loss	1.10	2.20	1.99	1.27	1.56	0.61	1.46
w/o CGComm Module and Alignment Loss	1.54	2.62	3.98	1.90	2.28	1.95	2.38

Table S16: Component-attribution analysis on regression datasets based on the relative RMSE increase (%) after removing each module.

Ablation variant	ESOL	FreeSolv	Lipophilicity	Avg. relative increase (%)
w/o Bipartite Graph Encoder	8.84	7.98	10.58	9.14
w/o Motif Transformer	16.38	9.73	3.53	9.88
w/o Molecular Descriptor	22.46	15.83	2.91	13.74
w/o CGComm Module	5.94	6.81	3.22	5.33
w/o Alignment Loss	12.90	8.63	3.07	8.20
w/o CGComm Module and Alignment Loss	12.17	9.15	4.45	8.59

12 Single-Granularity Contribution and Parameter-Matched Ablation Analysis

To further quantify the independent predictive contribution of each molecular granularity and examine whether the improvement of HMG-MRL simply arises from increased model capacity, we constructed four additional variants. Specifically, the three single-granularity variants, namely Bipartite Graph Encoder only, Motif Transformer only, and Global Attribute Encoder only, use only the atom–bond encoder, the motif encoder, or the molecular attribute encoder for property prediction, respectively. In addition, we introduced a parameter-matched variant, Bipartite Graph Encoder only (Param.-matched), by increasing the hidden dimension of the Bipartite Graph Encoder branch to make its trainable parameter count comparable to that of the full HMG-MRL model. The hidden dimensions and trainable parameter counts used for the parameter-matched variant are shown in Supplementary Table S17.

As shown in Supplementary Table S18, all single-granularity variants perform worse than the full HMG-MRL model, indicating that no individual granularity alone is sufficient to achieve the best predictive performance. Moreover, although the parameter-matched Bipartite Graph Encoder variant has a comparable number of trainable parameters to HMG-MRL, it still performs worse than HMG-MRL across all four datasets. These observations indicate that the performance gains of HMG-MRL mainly

arise from the complementary integration of atom–bond, motif, and molecular attribute information rather than from increased model capacity alone.

Table S17: Trainable-parameter comparison between the parameter-matched Bipartite Graph Encoder-only variant and the full HMG-MRL model.

Dataset	Hidden dimension	Bipartite Graph Encoder only (Param.-matched) #Params (M)	HMG-MRL #Params (M)
BACE	458	2.160	2.153
BBBP	345	1.235	1.234
ESOL	458	2.160	2.153
FreeSolv	345	1.235	1.234

Table S18: Performance comparison among single-granularity variants, the parameter-matched variant, and HMG-MRL. The best score in each column is highlighted in bold.

Model	BACE (ROC-AUC ↑)	BBBP (ROC-AUC ↑)	ESOL (RMSE ↓)	FreeSolv (RMSE ↓)
Bipartite Graph Encoder only	0.877±0.052	0.931±0.017	0.951±0.073	1.929±0.508
Motif Transformer only	0.854±0.065	0.907±0.032	1.271±0.604	2.208±0.819
Global Attribute Encoder only	0.862±0.061	0.917±0.014	0.868±0.028	1.853±0.575
Bipartite Graph Encoder only (Param.-matched)	0.883±0.051	0.928±0.025	0.935±0.092	1.976±0.606
**HMG-MRL**	**0.910±0.025**	**0.953±0.011**	**0.690±0.056**	**1.541±0.559**

13 Physicochemical Profiling of Isolated Non-Penetrating Clusters

To examine whether the isolated non-penetrating regions observed in the BBBP t-SNE visualization reflect chemically meaningful patterns related to BBB exclusion, we conducted a cluster-level physicochemical profiling analysis. Specifically, we focused on compounds labeled as non-penetrating (label 0) in the multi-granularity-fused representation space shown in Supplementary Fig. S2. A DBSCAN-based clustering analysis (Ester et al., 1996) was applied to these compounds in the t-SNE projection of the multi-granularity-fused representations, and clusters containing more than 20 compounds were retained for subsequent characterization. This procedure identified three major isolated non-penetrating regions, denoted as Region A, Region B, and Region C, as shown in Fig. S2.

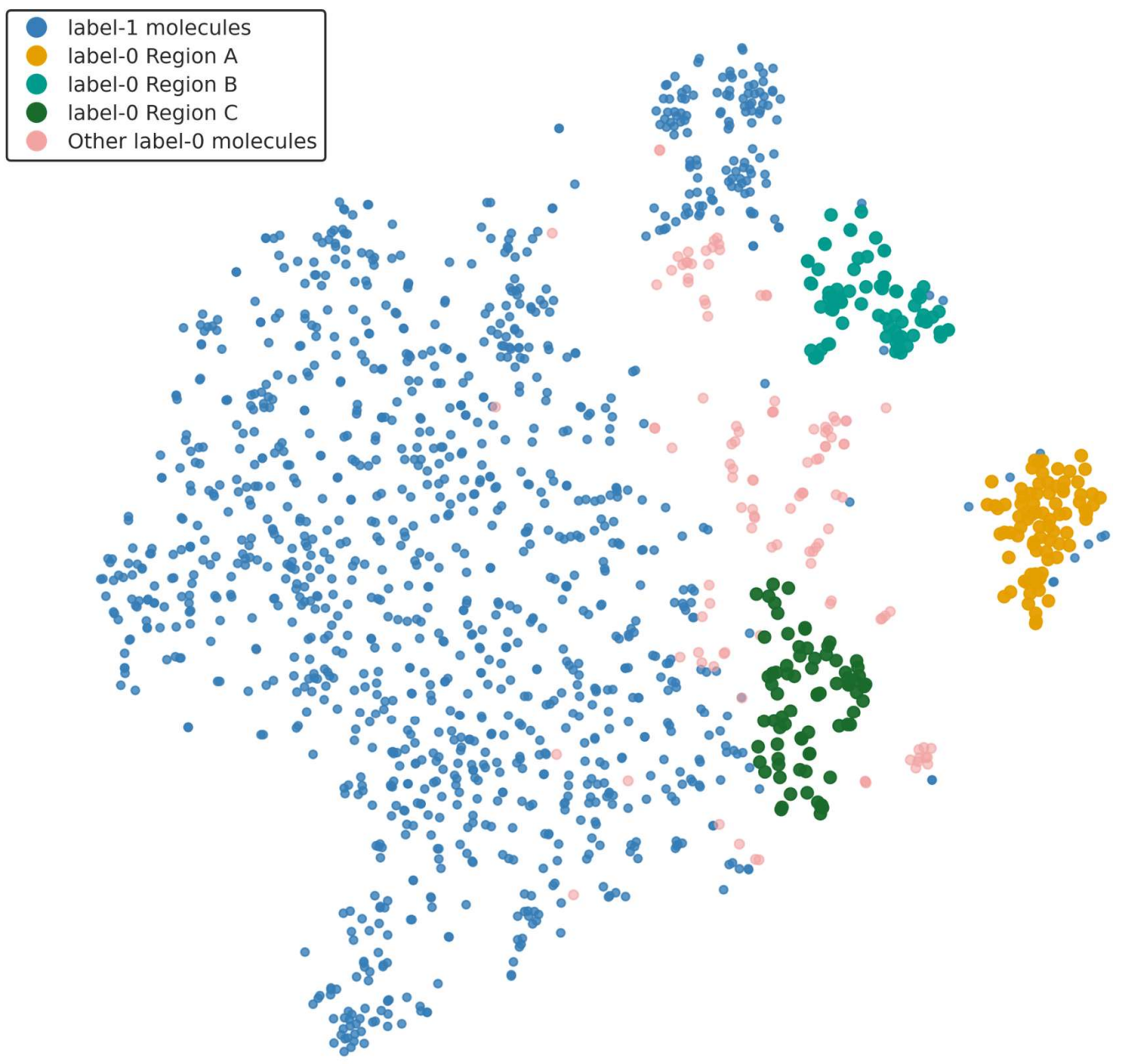

Figure S2: t-SNE visualization of isolated non-penetrating clusters on the BBBP dataset.

For each region, we calculated a set of BBB-permeability-related physicochemical descriptors using RDKit, including molecular weight (MolWt), MolLogP, topological polar surface area (TPSA), hydrogen bond donor count (HBD), and hydrogen bond acceptor count (HBA). These descriptors characterize molecular size, lipophilicity, polarity, and hydrogen-bonding capacity, which are closely associated with BBB penetration and barrier exclusion (Clark, 1999; van de Waterbeemd et al., 1998; Pajouhesh and Lenz, 2005). To provide a reference for comparison, all BBB-penetrating compounds (label 1) in the dataset were used as the BBB-penetrating reference group. The descriptor distributions are summarized as median and interquartile range (IQR) in Supplementary Table S19.

As shown in Supplementary Table S19, the three isolated non-penetrating regions exhibit distinct physicochemical profiles. Region A contains 82 compounds and shows a high-polarity and hydrogen-bond-rich profile, with increased TPSA, HBD, and HBA, together with markedly reduced MolLogP compared with the BBB-penetrating reference group. This profile is consistent with physicochemical characteristics that are unfavorable for BBB penetration.

Region B contains 75 compounds and shows the most pronounced barrier-exclusion profile among the three regions. Compared with the BBB-penetrating reference group, Region B has substantially larger molecular weight, markedly increased TPSA, and higher hydrogen bond donor and acceptor counts. These characteristics indicate that Region B represents a size-expanded, highly polar, and hydrogen-bond-intensive non-penetrating subtype.

Region C contains 77 compounds and exhibits a more moderate but still interpretable physicochemical shift. Compared with the BBB-penetrating reference group, Region C shows increased TPSA, HBD, and HBA, together with reduced MolLogP. Although this shift is less extreme than that observed in Regions A and B, it still indicates a distinct non-penetrating subtype characterized by moderately increased polarity and reduced lipophilicity.

These results suggest that the isolated non-penetrating regions in the fused representation space are associated with distinct physicochemical patterns related to BBB exclusion. This analysis provides additional support that the multi-granularity-fused representation learned by HMG-MRL captures chemically meaningful patterns relevant to BBB permeability.

Table S19: Physicochemical profiles of major isolated non-penetrating clusters on the BBBP dataset. Values are reported as median (interquartile range).

**Group**	**MolWt**	**MolLogP**	**TPSA**	**HBD**	**HBA**
BBB-penetrating reference (label-1)	304.8 (140.2)	2.8 (2.3)	46.6 (44.5)	1.0 (2.0)	3.0 (3.0)
Region A (label-0)	440.5 (88.1)	0.4 (2.0)	135.5 (60.1)	3.0 (2.0)	7.0 (4.0)
Region B (label-0)	670.9 (297.6)	1.3 (3.1)	198.4 (46.3)	5.0 (1.0)	13.0 (5.0)
Region C (label-0)	248.3 (109.9)	1.7 (1.8)	66.5 (37.0)	2.0 (2.0)	4.0 (1.0)

14 Attention-Guided Masking Analysis

To examine the predictive relevance of the attention patterns learned by HMG-MRL, an attention-guided masking analysis was conducted on ClinTox, BACE, and FreeSolv. The analysis was performed at both the atom and motif granularities.

For each test molecule, atoms and motifs were ranked according to their learned attention weights. Three masking strategies were evaluated: top-attention masking, random masking, and bottom-attention masking. Top-attention masking masks the atoms or motifs with the highest attention weights, bottom-attention masking masks those with the lowest attention weights, and random masking masks randomly selected atoms or motifs at the same masking ratio. Two masking ratios, 10% and 30%, were considered. The trained HMG-MRL model was kept fixed, and the masked samples were directly evaluated using

the same metrics as in the main experiments. For classification datasets, performance degradation is reflected by a decrease in ROC-AUC; for the regression dataset, performance degradation is reflected by an increase in RMSE.

The results are reported in Supplementary Tables S20 and S21. Across the three datasets, top-attention masking consistently caused the largest performance degradation. Under 10% motif masking, the ROC-AUC on ClinTox decreased from 0.947 to 0.835 when top-attention motifs were masked, compared with 0.925 under random masking and 0.945 under bottom-attention masking. On BACE, top-attention motif masking reduced ROC-AUC to 0.823, whereas random and bottom-attention masking resulted in ROC-AUC values of 0.857 and 0.907, respectively. On FreeSolv, top-attention motif masking increased RMSE from 1.541 to 2.200, which was larger than the changes caused by random masking and bottom-attention masking. Similar trends were observed under the 30% masking setting.

These results indicate that the atoms and motifs assigned higher attention weights generally have stronger predictive relevance than randomly selected or low-attention components. This attention-guided masking analysis supports the reliability of the learned atom- and motif-level attention patterns in identifying molecular components that contribute to HMG-MRL predictions.

Table S20: Attention-guided masking analysis with 10% masking on ClinTox, BACE, and FreeSolv. The best score in each row is highlighted in bold.

Dataset	HMG-MRL	Atom-level masking			Motif-level masking		
		Top masking	Random masking	Bottom masking	Top masking	Random masking	Bottom masking
ClinTox (ROC-AUC)	**0.947±0.005**	0.858±0.076	0.935±0.009	0.946±0.006	0.835±0.053	0.925±0.026	0.945±0.006
BACE (ROC-AUC)	**0.910±0.025**	0.887±0.046	0.895±0.033	0.905±0.029	0.823±0.093	0.857±0.057	0.907±0.022
FreeSolv (RMSE)	**1.541±0.559**	1.779±0.583	1.590±0.545	1.565±0.543	2.200±0.787	1.815±0.551	1.723±0.535

Table S21: Attention-guided masking analysis with 30% masking on ClinTox, BACE, and FreeSolv. The best score in each row is highlighted in bold.

Dataset	HMG-MRL	Atom-level masking			Motif-level masking		
		Top masking	Random masking	Bottom masking	Top masking	Random masking	Bottom masking
ClinTox (ROC-AUC)	**0.947±0.005**	0.836±0.090	0.912±0.022	0.941±0.007	0.792±0.087	0.884±0.037	0.934±0.006
BACE (ROC-AUC)	**0.910±0.025**	0.849±0.079	0.878±0.044	0.893±0.031	0.787±0.137	0.794±0.098	0.892±0.034
FreeSolv (RMSE)	**1.541±0.559**	1.980±0.688	1.692±0.617	1.641±0.567	2.278±0.827	2.207±0.837	1.927±0.662

References

A. P. Bento, A. Hersey, E. Félix, et al. An open source chemical structure curation pipeline using RDKit. *Journal of Cheminformatics*, 12(1):51, 2020.

H. Cai, H. Zhang, D. Zhao, et al. FP-GNN: a versatile deep learning architecture for enhanced molecular property prediction. *Briefings in Bioinformatics*, 23:bbac408, 2022.

J. Chen, S. Zheng, Y. Song, et al. Learning attributed graph representation with communicative message passing transformer. In *Proceedings of the Thirtieth International Joint Conference on Artificial Intelligence*, pages 2242–2248, 2021.

D. E. Clark. Rapid calculation of polar molecular surface area and its application to the prediction of transport phenomena. 2. prediction of blood-brain barrier penetration. *Journal of Pharmaceutical Sciences*, 88:815–821, 1999.

J. Degen, C. Wegscheid-Gerlach, A. Zaliani, et al. On the art of compiling and using 'drug-like' chemical fragment spaces. *ChemMedChem*, 3:1503–1507, 2008.

M. Ester, H.-P. Kriegel, J. Sander, and X. Xu. A density-based algorithm for discovering clusters in large spatial databases with noise. pages 226–231, 1996.

S. Han, H. Fu, Y. Wu, et al. HimGNN: a novel hierarchical molecular graph representation learning framework for property prediction. *Briefings in Bioinformatics*, 24:bbad305, 2023.

T.-H. Nguyen-Vo, T. T. Do, and B. P. Nguyen. ResGAT: Residual graph attention networks for molecular property prediction. *Memetic Computing*, 16:491–503, 2024.

H. Pajouhesh and G. R. Lenz. Medicinal chemical properties of successful central nervous system drugs. *NeuroRx*, 2:541–553, 2005.

Y. Song, S. Zheng, Z. Niu, et al. Communicative representation learning on attributed molecular graphs. In *Proceedings of the Twenty-Ninth International Joint Conference on Artificial Intelligence*, pages 2831–2838, 2020.

H. van de Waterbeemd, G. Camenisch, G. Folkers, J. R. Chretien, and O. A. Raevsky. Estimation of blood-brain barrier crossing of drugs using molecular size and shape, and h-bonding descriptors. *Journal of Drug Targeting*, 6:151–165, 1998.

Z. Wu, B. Ramsundar, E. N. Feinberg, et al. MoleculeNet: a benchmark for molecular machine learning. *Chemical Science*, 9:513–530, 2017.

Z. Wu, J. Wang, H. Du, et al. Chemistry-intuitive explanation of graph neural networks for molecular property prediction with substructure masking. *Nature Communications*, 14:2585, 2023.

Z. Xiong, D. Wang, X. Liu, et al. Pushing the boundaries of molecular representation for drug discovery with graph attention mechanism. *Journal of Medicinal Chemistry*, 63:8749–8760, 2020.

K. Yang, K. Swanson, W. Jin, et al. Analyzing learned molecular representations for property prediction. *Journal of Chemical Information and Modeling*, 59:3370–3388, 2019.

X. Zhao, S. Zhang, T. Zhang, et al. Molecular property prediction based on improved graph transformer network and multitask joint learning strategy. *Journal of Chemical Information and Modeling*, 65: 10561–10572, 2025.
